# Adaptive robust sparse representation for face recognition based on weighted and fusion dictionary

**DOI:** 10.1371/journal.pone.0351984

**Published:** 2026-06-26

**Authors:** Changming Song, Yang Zhou, Wenguang Ji

**Affiliations:** 1 School of Mathematics and Information Science, Zhengzhou Shengda University, China; 2 Samsung Semiconductor (China) Research & Development Co., Ltd, Beijing Branch, China; Northwest Normal University, CHINA

## Abstract

We propose a new model for face recognition under insufficient sampling conditions in this paper. In the proposed method, we combine the fusion dictionary with nuclear norm regularization to preserve the details of the restored images, and adopt a Laplacian-uniform mixture function to fit the error distribution. Since the proposed model is convex and separable, we employ the classic alternating direction method of multipliers to solve it by introducing auxiliary variables to transform the original problem into the saddle point problem. Theoretically, we conduct the convergence analysis of the proposed numerical algorithm. Final experimental comparisons are provided to verify the satisfactory performance of the proposed model, which outperforms other related competitive methods in both recognition rate and the robustness.

## Introduction

Face recognition is characterized by straightforward data collection, distinctive features, and strong anticounterfeiting capabilities. In recent years, it has been widely used in identity verification, attendance systems, mobile payments, criminal investigation, and other fields. Traditional face recognition algorithms have achieved favorable results under ideal conditions. However, certain limitations still exist in face recognition under non-ideal conditions. Particularly when samples are insufficient, recognition accuracy is significantly compromised by factors such as noise and occlusion. This poses a challenge for face recognition, which demands higher performance than traditional methods can offer.

Among the traditional face recognition algorithms, the Nearest Neighbor [1] (NN) classifier, the Nearest Neighbor Feature Classifier [2–5] and the Linear Regression Classifier [6] (LRC) are widely used due to their simplicity. The implementation of these classifiers is based on evaluating the relationship between test and training samples. Departing from these approaches, Wright et al. proposed the Sparse Representation-Based Classification (SRC) algorithm [7]. In SRC, training images form a dictionary, and the sparse representation is utilized to classify test images. This enables more effective recognition of face images that are corrupted by noise or occlusion. Subsequently, Zhang et al. proposed a Collaborative Representation Classification algorithm with Regularized Least Squares (CRC_RLS) [8]. Compared with SRC, this method achieves better recognition accuracy while effectively reducing computational complexity. Later, Zuo et al. proposed a face recognition algorithm based on $l_{p}$ norm sparse coding (SRC-P) [9] by employing the $l_{p}$ norm(0<p<1) to solve the sparse representation model. While the aforementioned models emphasize sparsity, they overlook the correlation information between images. To address this issue, Wang et al. proposed an Adaptive Sparse Representation Classification (ASRC) model [10], which leverages both sparsity and correlation information by considering both encoding sparsity and the nuclear norm of dictionaries. Consequently, this model adaptively benefits from the respective advantages of the $l_{\text{1}}$ norm and $l_{\text{2}}$ norm.

However, the effectiveness of the aforementioned methods significantly degrades in face recognition tasks with insufficient sampling. To address this issue, Deng et al. proposed the Extended Sparse Representation-Based Classification (ESRC) [11], which effectively mitigates the problem of low recognition rates under limited samples. Subsequently, Deng et al. developed the Superposed Linear Representation Classifier (SLRC) [12] by constructing a dictionary from a class-center matrix and an extended within-class variation matrix, thereby enhancing the generalization capability of collaborative representation. Furthermore, Yang et al. introduced a Robust Sparse Coding (RSC) model [13] that employs the maximum likelihood estimation principle to solve the sparse coding problem, reducing the model’s sensitivity to outliers. In recent years, further investigations have been conducted on sparse representation-based face recognition [14–29]. The latest research indicates that face recognition under complex conditions, such as noise and occlusion, remains a significant and worthwhile challenge, particularly in scenarios with insufficient sampling.

To further enhance the face recognition rate under conditions of under-sampling, occlusion, and noise, this paper comprehensively considers for the synergistic effects of image correlation, dictionary completeness, and algorithm robustness within a sparse representation framework. The main contributions of this work are summarized as follows:

(1) We propose a novel adaptive robust face recognition algorithm based on a weighted and fused dictionary. This method integrates the adaptive sparse representation classification framework with a weighted matrix and the concept of superposed linear representation, effectively enhancing the recognition accuracy and stability.
(2) The proposed algorithm is validated through extensive experiments on public face datasets. Results demonstrate that it outperforms several existing state-of-the-art methods, including NN, LRC, SRC, CRC_RLS, SLRC, ASRC, and RSC.

### Related work

In this section, we briefly survey previous work on image representation, concentrating particularly on sparse coding based approaches for face recognition.

Denote the training samples dataset of k th class as $A=[A_{\text{1}},A_{\text{2}},\ldots ,A_{i},\ldots ,A_{k}]\in R^{m\times n}$, where the submatrix $A_{i}\in R^{m\times n_{i}}$ is the sample image of class i th, and $n=\overset{k}{\underset{i=1}{\sum }}n_{i}$. Given a query sample $y\in R^{m}$, the recognition task is to determine which class y belongs to in the training sample matrix A, i.e.,


$$\begin{array}{c} y=Ax, \end{array}$$ (1)

where $x=[0,\ldots ,0,x_{i,1},x_{i,2},\ldots ,x_{i,n_{i}},0,\ldots ,0{]}^{T}\in R^{n}$ is the sparse coefficient vector. Most elements are zero in the sparse coefficient vector, except for those related to class i. That is, the non-zero entries in x are only those corresponding to training samples from the same class as y; coefficients from other classes are zero. This property allows the recognition task to be solved by finding the sparsest representation of y over A.

When sparse representation is initially used to describe face images, the following minimization objective function is adopted, i.e.,


$$\begin{array}{c} \underset{x}{min}{\|x\|}_{\text{0}}\text{s.t.}y=Ax, \end{array}$$ (2)

The above $l_{\text{0}}$ -norm minimization problem obviously exhibits sparsity, but it is an NP-hard problem. Researchers usually use the $l_{\text{1}}$ -norm that is closest to the $l_{\text{0}}$ -norm for sparse constraints under certain conditions, i.e.,


$$\begin{array}{c} \underset{x}{min}{\|x\|}_{\text{1}}\text{s.t.}y=Ax, \end{array}$$ (3)

In order to deal with the noise and avoid the non-zero terms of the sparse coefficient vector that are not related to the test sample, we add error constraints on the basis of the above model. Therefore, the above formulation is optimized to


$$\begin{array}{c} \underset{x}{min}{\|x\|}_{\text{1}}\text{s.t.}{\|y-Ax\|}_{\text{2}}\leq \varepsilon , \end{array}$$ (4)

where ε>0 is a given tolerance. The above equation can then be written as


$$\begin{array}{c} \hat{x}\text{= arg}\underset{x}{min}\overset{\text{2}}{\underset{\text{2}}{\|y-Ax\|}}+\lambda {\|x\|}_{\text{1}}, \end{array}$$ (5)

The first term ${\|y-Ax\|}_{\text{2}}$ in the above formula is the fidelity term, which represents the reconstruction error between the test image and the reconstructed image. The second term ${\|x\|}_{\text{1}}$ is the regularization term, which represents the sparsity of the coefficient. The parameter λ serves as a trade-off parameter that balances the fidelity term and the regularization term and it is also known as the regularization parameter. In the ideal case, a sample y in class k is selected for testing, then the non-zero terms in the regularization term x correspond to the entries in class k associated with the test sample. Due to the similarity of face images and the varying degrees of errors generated in face image processing, the non-zero terms in x that are unrelated to the test sample may also appear in the k -th class samples.

The characteristic function of class i samples is defined as $\delta_{i}:R^{n}\to R^{n}$, where $\delta_{i}\left(\hat{x}\right)\in R^{n}$ represents the coefficient vector of class i samples in $\hat{x}$. The test sample $\hat{y}=A\delta_{i}\left(\hat{x}\right)$ can be approximated based on the sparse representation of the training samples, and then classified according to the estimated residual $r_{i}\left(y\right)$ between the test sample $\hat{y}$ and original test sample y. Finally, the test sample y is assigned to the class with the smallest approximation residual, and then its mathematical representation is


$$\begin{array}{c} \underset{i}{min}\;r_{i}\left(y\right)={\|y-A\delta_{i}\left(\mathrm{\backslash stackrel}\wedge x\right)\|}_{\text{2}}\;i=1,2,\text{\textbackslash{}ldots},k \end{array}$$ (6)

The above SRC algorithm [2–7] relies on sufficient samples and precise alignment. Its performance deteriorates with insufficient dictionary atoms, highly correlated samples, or significant noise. Several studies have addressed these limitations. Among them, Zhang et al. proposed Collaborative Representation-based Classification (CRC), which utilizes regularized least squares with an $l_{\text{2}}$ -norm constraint on the representation coefficients. They argued that the collaborative representation mechanism itself, rather than the $l_{\text{1}}$ -norm-induced sparsity, is the primary contributor to the model’s effectiveness. The objective function is defined as follows:


$$\begin{array}{c} \underset{x}{min}{\|x\|}_{\text{2}}\text{s.t.}{\|y-Ax\|}_{\text{2}}\leq \varepsilon , \end{array}$$ (7)

Based on this foundation, Wang et al. introduced a trace norm constraint on the representation vector and proposed Adaptive Sparse Representation-Based Classification (ASRC), which adaptively integrates both $l_{\text{1}}$ norm and $l_{\text{2}}$ norm sparsity. However, ASRC remains sensitive to insufficient sampling. To address this issue, Deng et al. developed Extended Sparse Representation-Based Classification (ESRC), which augments the dictionary with intra-class variation bases. Further advancing this line of work, Deng et al. also proposed SLRC, representing a test image via a class-centered matrix and an intra-class variation matrix. While these methods have achieved some success, room for improvement remains. Yang et al. addressed the sparse coding problem by applying the principle of maximum likelihood estimation and proposed the Robust Sparse Coding (RSC) model, which reduced the model’s sensitivity to outliers. Subsequently, Dong et al. introduced the Low-Rank Laplacian-Uniform Mixture Model (LR-LUM), also based on MLE, to improve the error modeling capability. This method demonstrates strong robustness in the presence of noise in face recognition.

To address the limitations of existing methods, we propose a novel sparse representation approach termed ASRC-WFD. Building upon the adaptive sparse representation framework, our method incorporates an extended dictionary and a weight function, enhancing its robustness in scenarios characterized by insufficient sampling and diverse types of noise. The detailed formulation of ASRC-WFD is provided in next section.

### Adaptive sparse representation classification based weighted and fusion dictionary

This section presents our adaptive sparse representation model with a weighted and fusion dictionary in detail and derives its solution. We formally refer to the resulting face recognition framework as ASRC-WFD.

### Our model

In order to construct a more robust model for sparse coding of face images, in this paper we propose to find a maximum likelihood estimation (MLE) solution [30] for the coding coefficients. Assume that the elements $e_{i}$ of the coding residual $e=y-Ax=[e_{\text{1}};e_{\text{2}};\ldots ;e_{i};\ldots ;e_{m}]$ are independently and identically distributed according to some probability density function (PDF) $f\left(e_{i}\right)$. Without considering the sparsity constraint of x, the likelihood of the estimator is$L\left(e\right)=L(e_{\text{1}},e_{\text{2}},\ldots ,e_{m})=\overset{m}{\underset{i=1}{\prod }}f\left(e_{i}\right)$, and MLE aims to maximize this likelihood function or, equivalently, minimize theobjective function: $F\left(e\right)=\overset{m}{\underset{i=1}{\sum }}\rho \left(e_{i}\right)$, where $\rho \left(e_{i}\right)=-ln\left(f\left(e_{i}\right)\right)$. We can approximate F(e) by its first-order Taylor expansion in the neighborhood of $\rho \left(\overset{t}{\underset{i}{e}}\right)$,


$$\begin{array}{c} F\left(e\right)=\sum_{i}\left[\rho \left(\left|\overset{t}{\underset{i}{e}}\right|\right)+\rho^{\prime }\left(\left|\overset{t}{\underset{i}{e}}\right|\right)\left(\left|e_{i}\right|-\left|\overset{t}{\underset{i}{e}}\right|\right)\right], \end{array}$$ (8)

where $\rho^{\prime }\left(\left|\overset{t}{\underset{i}{e}}\right|\right)$ is the derivative of $\rho \left(\left|\overset{t}{\underset{i}{e}}\right|\right)$. In order to better describe random errors in challenging situations, we adopt a Laplacian-uniform mixed (LUM) function to fit the error distribution, which can be expressed as


$$\begin{array}{c} f\left(e_{i}\right)=\alpha \left(exp\left(-\left|e_{i}\right|/b\right)+c\right), \end{array}$$ (9)

where b>0 corresponds to the scale of the Laplacian component and c>0 corresponds to a uniform distribution, α>0 is a distribution normalization factor. Denote by $f^{\prime }\left(e_{i}\right)$ the derivative of $f\left(e_{i}\right)$, and then $f^{\prime }\left(e_{i}\right)\text{=}-\left(\alpha /b\right)\left(exp\left(-\left|e_{i}\right|/b\right)\right)$. Then (8) can be rewritten as


$$\begin{array}{c} F\left(e\right)=\sum_{i}\left[\rho \left(\left|\overset{t}{\underset{i}{e}}\right|\right)+\frac{\text{1}}{\text{b}}\cdot \frac{e^{-\left|\overset{t}{\underset{i}{e}}\right|/b}}{e^{-\left|\overset{t}{\underset{i}{e}}\right|/b}+c}\left(\left|e_{i}\right|-\left|\overset{t}{\underset{i}{e}}\right|\right)\right], \end{array}$$ (10)

The constant term constraint in the above equation can be omitted,


$$\begin{array}{c} F\left(e\right)=\sum_{i}\left[\frac{e^{-\left|\overset{t}{\underset{i}{e}}\right|/b}}{e^{-\left|\overset{t}{\underset{i}{e}}\right|/b}+c}e_{i}\right]\text{=}\sum_{i}\left|\overset{t}{\underset{i}{w}}e_{i}\right|={\|We\|}_{\text{1}}, \end{array}$$ (11)

The i -th element of the diagonal matrix W is denoted by $\overset{t}{\underset{i}{w}}=\frac{exp\left(-\left|\overset{t}{\underset{i}{e}}\right|/b\right)}{exp\left(-\left|\overset{t}{\underset{i}{e}}\right|/b\right)+c}$. So F(e) term becomes a reweighted $l_{\text{1}}$ -norm constraint. Furthermore, the objective function is expressed in the following form,


$$\begin{array}{c} \underset{x}{min}{\|We\|}_{\text{1}}+\lambda {\|x\|}_{\text{1}}\text{s.t.}y-Ax=e, \end{array}$$ (12)

where λ>0 is the regularization parameter. The regular term ${\|x\|}_{\text{1}}$ is to prevent overfitting of the model.

In the fidelity term ${\|We\|}_{\text{1}}$, we incorporate a weighted $l_{\text{1}}$ norm constraint to account for potential errors in the image data. To further enhance the model’s performance, additional constraints are introduced to the regularization term. As demonstrated by Wang et al. [10], while sparsity effectively selects relevant samples, the correlation structure helps capture the underlying relationships between test and training samples. To simultaneously leverage both sparsity and correlation, we impose nuclear norm constraints on both the dictionary and the representation vectors. Consequently, the objective function (12) is reformulated as follows with the application of a nuclear norm constraint to the regularization term:


$$\begin{array}{c} \underset{x}{min}{\|We\|}_{\text{1}}+\lambda {\|ADiag\left(x\right)\|}_{*}\text{s.t.}y-Ax=e, \end{array}$$ (13)

where ${\|ADiag\left(x\right)\|}_{*}$ is the correlation regularized. We make the following two extreme inferences from this model.

First, assume that the samples are unrelated and the columns of the dictionary matrix A are orthogonal, $A^{T}A=I$. Then equation (13) will be converted into the following problem:


$$\begin{array}{c} \begin{array}{c} \;\underset{x}{min}{\|We\|}_{\text{1}}+\lambda {\|ADiag\left(x\right)\|}_{*}\\ =\underset{x}{min}{\|We\|}_{\text{1}}+\lambda \cdot Tr{\left[{\left(ADiag\left(x\right)\right)}^{T}\left(ADiag\left(x\right)\right)\right]}^{\frac{\text{1}}{\text{2}}}\\ =\underset{x}{min}{\|We\|}_{\text{1}}+\lambda \cdot Tr{\left[{\left(Diag\left(x\right)\right)}^{T}\left(Diag\left(x\right)\right)\right]}^{\frac{\text{1}}{\text{2}}}\\ =\underset{x}{min}{\|We\|}_{\text{1}}+\lambda {\|x\|}_{\text{1}} \end{array} \end{array}$$ (14)

In this case, it is simplified to the problem of Equation (12), which indicates that the $l_{\text{1}}$ norm sparse constraint in the regular term takes into account the non-correlation between the training samples.

Second, we assume that images of different subjects look similar to $a_{i}$, and then we have $A=a_{i}{\text{1}}^{T}$ and $A^{T}A=\text{1}{\text{1}}^{T}$ (1 is a vector of size n,where all the elements are one). Then, equation (13) is converted to


$$\begin{array}{c} \begin{array}{c} \;\underset{x}{min}{\|We\|}_{\text{1}}+\lambda {\|ADiag\left(x\right)\|}_{*}\\ \text{=\textasciitilde{}}\underset{x}{min}{\|We\|}_{\text{1}}+\lambda {\|a_{i}x^{T}\|}_{*}\\ =\underset{x}{min}{\|We\|}_{\text{1}}+\lambda {\|a_{i}\|}_{\text{2}}{\|x\|}_{\text{2}}\\ =\underset{x}{min}{\|We\|}_{\text{1}}+\lambda {\|x\|}_{\text{2}} \end{array} \end{array}$$ (15)

where the $l_{\text{2}}$ norm sparse constraint of the regularization term can be used to process highly correlated images. The above hypothetical inference precisely indicates that the model effectively harnesses both $l_{\text{1}}$ and $l_{\text{2}}$ norm constraints by leveraging the inherent correlation among sample images. In practical applications, face images are neither perfectly aligned nor entirely independent. The introduced nuclear norm constraint successfully balances this complex dependency.

In addition to incorporating weight constraints for random errors and modeling sparsity and correlation, we address the coding errors arising from insufficient or unrepresentative training samples. A literature review demonstrates that the SLRC algorithm exhibits notable stability under such sampling conditions. This finding motivates us to decompose the training sample dictionary into a class-centered matrix


$$\begin{array}{c} P=[c_{\text{1}},\ldots ,c_{i},\ldots ,c_{k}]\in R^{m\times k}, \end{array}$$ (16)

and an intra-class variation matrix


$$\begin{array}{c} V=[A_{\text{1}}-c_{\text{1}}\overset{T}{\underset{\text{1}}{e}},\ldots ,A_{i}-c_{i}\overset{T}{\underset{i}{e}},\ldots ,A_{k}-c_{k}\overset{T}{\underset{k}{e}}]\in R^{m\times n}, \end{array}$$ (17)

enhancing robustness against limited data, where $e_{i}=[1,1,\ldots ,1{]}^{T}\in R^{n_{i}\times 1}$, $c_{i}$ is the class centroid of class i. Then, the class-center matrix and the intra-class variation matrix are fused as a new fusion dictionary. Then the new fusion dictionary can be fused by the class-center matrix and the in-class variation matrix as follows:


$$\begin{array}{c} F=\left[P,V\right]\in R^{m\times \left(k+n\right)}, \end{array}$$ (18)

Based on the above analysis, we propose an adaptive robust sparse representation model using weighted and fused dictionaries.


$$\begin{array}{c} \underset{x_{p},x_{v}}{min}{\|W\left(y-\left[P,V\right]\left[\begin{array}{c} @c@x_{p}\\ x_{v} \end{array}\right]\right)\|}_{\text{1}}+\lambda {\|\left[P,V\right]Diag\left[\begin{array}{c} @c@x_{p}\\ x_{v} \end{array}\right]\|}_{*}, \end{array}$$ (19)

This mixed model is motivated by the key error sources in face recognition: random post-processing noise, reconstruction inaccuracy, alignment perturbations, and insufficient sampling. Our approach integrates a weighted $l_{\text{1}}$ norm constraint to model errors, a fused dictionary to compensate for limited data, and a nuclear norm to improve correlation learning, collectively enhancing robustness and accuracy.

### Optimization

To solve problem (19), we first convert it to the following equivalent optimization problem:


$$\begin{array}{c} \underset{x}{min}{\|W\left(y-Fx\right)\|}_{\text{1}}+\lambda {\|FDiag\left(x\right)\|}_{*}, \end{array}$$ (20)

where F=[P,V] and $x=\left[x_{p};x_{v}\right]$. Inspired by the optimization method used in [21] and [22] we adopt alternating direction method of multipliers (ADMM) [31] to solve problem (20). We first convert it to the following equivalent problem:


$$\begin{array}{c} \underset{\hat{e},J,x}{min}{\|\hat{e}\|}_{\text{1}}\text{+}\lambda {\|J\|}_{*}\text{s.t.}\hat{e}=We=W\left(y-Fx\right)\text{,}J=FDiag\left(x\right)\; \end{array}$$ (21)

Problem (21) can be solved by solving the following augmented Lagrange multiplier problem:


$$\begin{array}{c} L\left(J,x,\hat{e},y_{\text{1}},Y_{\text{2}}\right)={\|\hat{e}\|}_{\text{1}}\text{+}\lambda {\|J\|}_{*}+\langle y_{\text{1}},\text{\textbackslash{}hspace\{0.17em}\}\hat{e}-W\left(y-Fx\right)\rangle +\langle Y_{\text{2}},\text{\textbackslash{}hspace\{0.17em}\}J-FDiag\left(x\right)\rangle +\frac{\mu }{\text{2}}\left(\overset{\text{2}}{\underset{\text{2}}{\|\hat{e}-W\left(y-Fx\right)\|}}\text{+}\overset{\text{2}}{\underset{F}{\|J-FDiag\left(x\right)\|}}\right) \end{array}$$ (22)

where $y_{\text{1}}$ and $Y_{\text{2}}$ are Lagrange multipliers and μ>0 is a parameter. In general, obtaining optimal solution of (22) is equivalent to finding a saddle point $\left(J^{*},x^{*},{\hat{e}}^{*},{y_{\text{1}}}^{*},{Y_{\text{2}}}^{*}\right)$ of the min-max problem


$$\begin{array}{c} \underset{J,x,\hat{e}}{min}\;\underset{y_{\text{1}},Y_{\text{2}}}{max}L\left(J,x,\hat{e},y_{\text{1}},Y_{\text{2}}\right), \end{array}$$ (23)

Such that


$$\begin{array}{c} L\left(J^{*},x^{*},{\hat{e}}^{*},y_{\text{1}},Y_{\text{2}}\right)\leq L\left(J^{*},x^{*},{\hat{e}}^{*},{y_{\text{1}}}^{*},{Y_{\text{2}}}^{*}\right)\leq L\left(J,x,\hat{e},{y_{\text{1}}}^{*},{Y_{\text{2}}}^{*}\right) \end{array}$$ (24)

This optimization problem can be minimized with respect to J, x and $\hat{e}$, respectively, by fixing the other variables, and the updating $y_{\text{1}}$ and $Y_{\text{2}}$. As a result, the original problem is decomposed into several subproblems as follow:


$$\begin{array}{c} J^{t+1}\text{=}arg\underset{J}{min}\lambda {\|J\|}_{*}+\langle \overset{t}{\underset{\text{2}}{Y}},\text{\textbackslash{}hspace\{0.17em}\}J-FDiag\left(x^{t}\right)\rangle +\frac{\mu }{\text{2}}\overset{\text{2}}{\underset{F}{\|J-FDiag\left(x^{t}\right)\|}} \end{array}$$ (25)

$$\begin{array}{c} \begin{array}{c} x^{t+1}\text{=}arg\underset{x}{min}\langle \overset{t}{\underset{\text{1}}{y}},\text{\textbackslash{}hspace\{0.17em}\}{\hat{e}}^{t}-W\left(y-Fx\right)\rangle +\frac{\mu }{\text{2}}\overset{\text{2}}{\underset{\text{2}}{\|{\hat{e}}^{t}-W\left(y-Fx\right)\|}}\\ \text{+}\langle \overset{t}{\underset{\text{2}}{Y}},\text{\textbackslash{}hspace\{0.17em}\}J^{t+1}-FDiag\left(x\right)\rangle +\frac{\mu }{\text{2}}\overset{\text{2}}{\underset{F}{\|J^{t+1}-FDiag\left(x\right)\|}}, \end{array} \end{array}$$ (26)

$$\begin{array}{c} {\hat{e}}^{t+1}=arg\underset{\hat{e}}{min}{\|\hat{e}\|}_{\text{1}}+\langle \overset{t}{\underset{\text{1}}{y}},\text{\textbackslash{}hspace\{0.17em}\}\hat{e}-W\left(y-Fx^{t+1}\right)\rangle +\frac{\mu }{\text{2}}\overset{\text{2}}{\underset{\text{2}}{\|\hat{e}-W\left(y-Fx^{t+1}\right)\|}}, \end{array}$$ (27)

$$\begin{array}{c} \overset{t+1}{\underset{\text{1}}{y}}=\overset{t}{\underset{\text{1}}{y}}+\mu \left({\hat{e}}^{t\text{+}1}-W\left(y-Fx^{t+1}\right)\right), \end{array}$$ (28)

$$\begin{array}{c} \overset{t+1}{\underset{\text{2}}{Y}}=\overset{t}{\underset{\text{2}}{Y}}+\mu \left(J^{t+1}-FDiag\left(x^{t+1}\right)\right), \end{array}$$ (29)

where t denotes the iteration index in the algorithm.

First, we optimize problem (25) by solving the following subproblem:


$$\begin{array}{c} \begin{array}{c} J^{t+1}\text{= arg}\underset{J}{\text{min}}\frac{\lambda }{\mu }{\|J\|}_{*}+\frac{\text{1}}{\text{2}}\overset{\text{2}}{\underset{F}{\|J-\left(FDiag\left(x^{t}\right)-\frac{\overset{t}{\underset{\text{2}}{Y}}}{\mu }\right)\|}}\\ \text{= arg}\underset{J}{min}\eta {\|J\|}_{*}+\frac{\text{1}}{\text{2}}\overset{\text{2}}{\underset{F}{\|J-F_{J}\|}} \end{array} \end{array}$$ (30)

where the $\eta \text{=}\frac{\lambda }{\mu }$ and $F_{J}=FDiag\left(x^{t}\right)-\frac{\overset{t}{\underset{\text{2}}{Y}}}{\mu }$. Problem (30) is a nuclear norm minimization problem and a closed form solution can be obtained through singularity Value Threshold (SVT) algorithm [32]. Let $F_{J}\text{=}U\Sigma V^{T}$ be the SVD of $F_{J}$, where Σ is singularity value matrix of $F_{J}$, U and V are corresponding orthogonal matric. Then, the optimal solution can be obtained as follows:


$$\begin{array}{c} J^{\text{t+1}}=US_{\eta }\left(\Sigma \right)V^{T} \end{array}$$ (31)

where the SVT operator is defined as follows $S_{\eta }\left(\Sigma \right)=diag\left(max\left(\Sigma_{i,i}-\eta ,0\right)\right)$.

Second, we consider how to solve the problem Eq.(26). Eq.(26) is equivalent to


$$\begin{array}{c} \begin{array}{c} x^{t+1}\text{=arg}\underset{x}{\text{min}}\langle \overset{t}{\underset{\text{1}}{y}},WFx\rangle \text{+}\frac{\mu }{\text{2}}\overset{\text{2}}{\underset{\text{2}}{\|WFx\|}}+\mu \langle {\hat{e}}^{t}-Wy,WFx\rangle \\ \;-\langle \overset{t}{\underset{\text{2}}{Y}},FDiag\left(x\right)\rangle +\frac{\mu }{\text{2}}\overset{\text{2}}{\underset{F}{\|FDiag\left(x\right)\|}}-\mu \langle J^{t+1},FDiag\left(x\right)\rangle \\ \text{=arg}\underset{x}{\text{min}}\frac{\mu }{\text{2}}x^{T}\left(F^{T}W^{T}WF+Diag\left(diag\left(F^{T}F\right)\right)\right)x\\ \;-{\left(-F^{T}W^{T}\overset{t}{\underset{\text{1}}{y}}+\mu F^{T}W^{T}\left(Wy-{\hat{e}}^{t}\right)+diag\left(F^{T}\overset{t}{\underset{\text{2}}{Y}}+\mu F^{T}J^{t+1}\right)\right)}^{T}x \end{array} \end{array}$$ (32)

The above problem can be easily solved by


$$\begin{array}{c} x^{t+1}=BF^{T}W^{T}\left(Wy-{\hat{e}}^{t}-\frac{\overset{t}{\underset{\text{1}}{y}}}{\mu }\right)+Bdiag\left(F^{T}\left(\frac{\overset{t}{\underset{\text{2}}{Y}}}{\mu }+J^{t+1}\right)\right), \end{array}$$ (33)

where $B={\left(F^{T}W^{T}WF+Diag\left(diag\left(F^{T}F\right)\right)\right)}^{-1}$.

Third, we consider how to solve the problem Eq.(27). Eq.(27) is equivalent to solving the following problem:


$$\begin{array}{c} \begin{array}{c} {\hat{e}}^{t+1}\text{= arg}\underset{\hat{e}}{min}\frac{\text{1}}{\mu }{\|\hat{e}\|}_{\text{1}}+\frac{\text{1}}{\text{2}}\overset{\text{2}}{\underset{\text{2}}{\|\hat{e}-\left(W\left(y-Fx^{t+1}\right)-\frac{\overset{t}{\underset{\text{1}}{y}}}{\mu }\right)\|}}\\ \text{ = arg}\underset{\hat{e}}{min}\frac{\text{1}}{\mu }{\|\hat{e}\|}_{\text{1}}+\frac{\text{1}}{\text{2}}\overset{\text{2}}{\underset{\text{2}}{\|\hat{e}-W_{\hat{e}}\|}} \end{array} \end{array}$$ (34)

where $W_{\hat{e}}=W\left(y-Fx^{t+1}\right)-\frac{\overset{t}{\underset{\text{1}}{y}}}{\mu }$. The above problem can be obtained a closed form solution by soft-thresholding operator as follows:


$$\begin{array}{c} {\hat{e}}^{t+1}=soft\left(W_{\hat{e}},\left(1/\mu \right)\right), \end{array}$$ (35)

The soft-thresholding operator is defined as follows:


$$\begin{array}{c} soft{\left(x,\alpha \right)}_{i}=sgn\left(x_{i}\right)\cdot max\left(\left|x_{i}\right|-\alpha ,0\right), \end{array}$$ (36)

where $sgn\left(x_{i}\right)$ is sign function, .

We set the original residual to the minimum to converge to the optimal solution through the ADMM problem. Therefore, the constraints are proposed as follows: ${\|W\left(y-Fx\right)-\hat{e}\|}_{\infty }\leq \varepsilon_{\text{1}}$ and ${\|J-FDiag\left(x\right)\|}_{\infty }\leq \varepsilon_{\text{2}}$, where $\varepsilon_{\text{1}}$ and $\varepsilon_{\text{2}}$ are a small positive scalar. Convergence is achieved when the difference in the weights between adjacent iterations is sufficiently small. Specifically, we stop the iteration if the following condition holds: ${\|W^{t}-W^{t-1}\|}_{\text{2}}/{\|W^{t-1}\|}_{\text{2}}<\varepsilon_{\text{3}}$, where $\varepsilon_{\text{3}}$ is a small positive scalar.

Based on the above analysis, the model optimization process is summarized into algorithm 1:

**Algorithm 1: The iterative optimization algorithm for the proposed model** $min{\|W\left(y-Fx\right)\|}_{\text{1}}+\lambda {\|FDiag\left(x\right)\|}_{*}$

Input: A test image $y\in R^{m}$, the fusion dictionary $F=\left[P,V\right]\in R^{m\times \left(k+n\right)}$,

Output: the identity with the converged $W^{*}$ and $x^{*}=\left[\overset{*}{\underset{p}{x}};\overset{*}{\underset{v}{x}}\right]$,

(1)  Initialize t=0, $x^{t}=\left[\frac{\text{1}}{n},\frac{\text{1}}{n},\text{\textbackslash{}ldots},\frac{\text{1}}{n}\right]$, $e^{t}=\frac{\text{1}}{\text{2}}\left(y-Fx^{t}\right)$, $\overset{t}{\underset{\text{1}}{y}}=0$, $\overset{t}{\underset{\text{2}}{Y}}=0$

(2)  Repeat

(3)   Update the weight matrix W via (11)

(4)   repeat

(5)     Update J via (31)

(6)     Update x via (33)

(7)     Update $\hat{e}$ via (35)

(8)     Update $y_{\text{1}}$ and $Y_{\text{2}}$ via (28) and (29)

(9)     Update $\mu =min\left(\rho \mu ,\mu_{max}\right)$

(10)     t=t+1

(11)   until convergence or maximum iterations

(12)  until convergence or maximum iterations

### Convergence analysis

In this subsection, we aim to establish the convergence of Algorithm 1. To this end, we generally need some assumptions including that the objective function is the closed proper and convex and the problem (23) has a saddle point. With these facts, we have the following convergence analysis.

**Theorem 1.** Let $\left(J^{*},x^{*},{\hat{e}}^{*},{y_{\text{1}}}^{*},{Y_{\text{2}}}^{*}\right)$ be a saddle point of the min-max problem (23), then the sequence $\left\{\left(J^{t\text{+1}},x^{t\text{+1}},{\hat{e}}^{t+1},{y_{\text{1}}}^{t+\text{1}},{Y_{\text{2}}}^{t\text{+1}}\right)\right\}$ generated by Algorithm 1 is convergent.

*Proof.* By the definition of the saddle point of the Lagrangian function (23), we can deduce that ${\hat{e}}^{*}\text{=}W\left(y-Fx^{*}\right)$, $J^{*}\text{=}FDiag\left(x^{*}\right)$. This fact coupling with (28) and (29) leads to


$$\begin{array}{c} \left\{ \begin{array}{c} @c@\overset{*}{\underset{\text{1}}{y}}=\overset{*}{\underset{\text{1}}{y}}+\mu \left({\hat{e}}^{*}-W\left(y-Fx^{*}\right)\right)\\ \overset{*}{\underset{\text{2}}{Y}}=\overset{*}{\underset{\text{2}}{Y}}+\mu \left(J^{*}-FDiag\left(x^{*}\right)\right) \end{array}\right. \end{array}$$ (37)

We set the relative errors by $\overset{t}{\underset{e}{J}}\text{=}J^{t}-J^{*}$, $\overset{t}{\underset{e}{x}}\text{=}x^{t}-x^{*}$, $\overset{t}{\underset{e}{\hat{e}}}\text{=}{\hat{e}}^{t}-{\hat{e}}^{*}$, $\overset{t}{\underset{1e}{y}}\text{=}\overset{t}{\underset{\text{1}}{y}}-\overset{*}{\underset{\text{1}}{y}}$, $\overset{t}{\underset{2e}{Y}}\text{=}\overset{t}{\underset{\text{2}}{Y}}-\overset{*}{\underset{\text{2}}{Y}}$ and then subtracting (37) from (28)- (29), we can obtain that


$$\begin{array}{c} \left\{ \begin{array}{c} @l@\overset{t+1}{\underset{1e}{y}}=\overset{t}{\underset{1e}{y}}+\mu \left(\overset{t+1}{\underset{e}{\hat{e}}}+WF\overset{t+1}{\underset{e}{x}}\right)\\ \overset{t+1}{\underset{2e}{Y}}=\overset{t}{\underset{2e}{Y}}+\mu \left(\overset{t+1}{\underset{e}{J}}-FDiag\left(\overset{t+1}{\underset{e}{x}}\right)\right) \end{array}\right. \end{array}$$ (38)

Squaring both sides of (38) then yields


$$\begin{array}{c} \left\{ \begin{array}{c} @l@\overset{\text{2}}{\underset{\text{2}}{\|\overset{t+1}{\underset{1e}{y}}\|}}=\overset{\text{2}}{\underset{\text{2}}{\|\overset{t}{\underset{1e}{y}}\|}}+2\mu \langle \overset{t}{\underset{1e}{y}},\overset{t+1}{\underset{e}{\hat{e}}}+WF\overset{t+1}{\underset{e}{x}}\rangle \text{+}\mu^{\text{2}}\overset{\text{2}}{\underset{\text{2}}{\|\overset{t+1}{\underset{e}{\hat{e}}}+WF\overset{t+1}{\underset{e}{x}}\|}}\\ \overset{\text{2}}{\underset{F}{\|\overset{t+1}{\underset{2e}{Y}}\|}}=\overset{\text{2}}{\underset{F}{\|\overset{t}{\underset{2e}{Y}}\|}}+2\mu \langle \overset{t}{\underset{2e}{Y}},\overset{t+1}{\underset{e}{J}}-FDiag\left(\overset{t+1}{\underset{e}{x}}\right)\rangle \text{+}\mu^{\text{2}}\overset{\text{2}}{\underset{F}{\|\overset{t+1}{\underset{e}{J}}-FDiag\left(\overset{t+1}{\underset{e}{x}}\right)\|}} \end{array}\right. \end{array}$$ (39)

Furthermore, we can equivalently reformulate them as


$$\begin{array}{c} \left\{ \begin{array}{c} @l@\frac{\text{1}}{2\mu }\left(\overset{\text{2}}{\underset{\text{2}}{\|\overset{t}{\underset{1e}{y}}\|}}-\overset{\text{2}}{\underset{\text{2}}{\|\overset{t+1}{\underset{1e}{y}}\|}}\right)=-\langle \overset{t}{\underset{1e}{y}},\overset{t+1}{\underset{e}{\hat{e}}}+WF\overset{t+1}{\underset{e}{x}}\rangle -\frac{\mu }{\text{2}}\overset{\text{2}}{\underset{\text{2}}{\|\overset{t+1}{\underset{e}{\hat{e}}}+WF\overset{t+1}{\underset{e}{x}}\|}}\\ \frac{\text{1}}{2\mu }\left(\overset{\text{2}}{\underset{F}{\|\overset{t}{\underset{2e}{Y}}\|}}-\overset{\text{2}}{\underset{F}{\|\overset{t+1}{\underset{2e}{Y}}\|}}\right)=-\langle \overset{t}{\underset{2e}{Y}},\overset{t+1}{\underset{e}{J}}-FDiag\left(\overset{t+1}{\underset{e}{x}}\right)\rangle -\frac{\mu }{\text{2}}\overset{\text{2}}{\underset{F}{\|\overset{t+1}{\underset{e}{J}}-FDiag\left(\overset{t+1}{\underset{e}{x}}\right)\|}} \end{array}\right. \end{array}$$ (40)

In order to obtain the monotonicity of convex function (25)-(27), the F(x)=H(x)+G(x) lemma is needed, please refer to Lemma 1 in Appendix B.

According to lemma 1 and the saddle point of Lagrangian function (22), we also get the monotonicity of the function as follow:


$$\begin{array}{c} \lambda \left({\|J\|}_{*}-{\|J^{*}\|}_{*}\right)+\langle \overset{*}{\underset{\text{2}}{Y}},J-J^{*}\rangle +\mu \langle J^{*}-FDiag\left(x^{*}\right),J-J^{*}\rangle \geq 0 \end{array}$$ (41)

$$\begin{array}{c} \begin{array}{c} \text{-}\langle Diag\left(F^{T}\overset{*}{\underset{\text{2}}{Y}}\right),x-x^{*}\rangle -\mu \langle Diag\left(F^{T}\left(J^{*}-FDiag\left(x^{*}\right)\right)\right),x-x^{*}\rangle \\ \text{+}\langle {\left(WF\right)}^{T}\overset{*}{\underset{\text{1}}{y}},x-x^{*}\rangle \text{+}\mu \langle {\left(WF\right)}^{T}\left({\hat{e}}^{*}-W\left(y-Fx^{*}\right)\right),x-x^{*}\rangle \geq 0 \end{array} \end{array}$$ (42)

$$\begin{array}{c} {\|\hat{e}\|}_{\text{1}}-{\|{\hat{e}}^{*}\|}_{\text{1}}+\langle \overset{*}{\underset{\text{1}}{y}},\hat{e}-{\hat{e}}^{*}\rangle +\mu \langle {\hat{e}}^{*}-W\left(y-Fx^{*}\right),\hat{e}-{\hat{e}}^{*}\rangle \geq 0 \end{array}$$ (43)

To solve the subproblem 26, we need to fix $J^{t}$， ${\hat{e}}^{t}$， ${y_{\text{1}}}^{t}$ and ${Y_{\text{2}}}^{t}$, then obtaining $x^{t\text{+1}}$ have


$$\begin{array}{c} \begin{array}{c} \text{-}\langle Diag\left(F^{T}\overset{t}{\underset{\text{2}}{Y}}\right),x-x^{t+1}\rangle -\mu \langle Diag\left(F^{T}\left(J^{t}-FDiag\left(x^{t\text{+}1}\right)\right)\right),x-x^{t+1}\rangle \\ \text{+}\langle {\left(WF\right)}^{T}\overset{t}{\underset{\text{1}}{y}},x-x^{t+1}\rangle \text{+}\mu \langle {\left(WF\right)}^{T}\left({\hat{e}}^{t}-W\left(y-Fx^{t+1}\right)\right),x-x^{t+1}\rangle \geq 0 \end{array} \end{array}$$ (44)

Taking $x\text{=}x^{t+1}$ in (42) and $x\text{=}x^{*}$ in (44) respectively, we add them together and take the aforementioned relative error into full account ($\overset{t}{\underset{e}{J}}\text{=}J^{t}-J^{*}$, $\overset{t}{\underset{e}{x}}\text{=}x^{t}-x^{*}$, $\overset{t}{\underset{e}{\hat{e}}}\text{=}{\hat{e}}^{t}-{\hat{e}}^{*}$, $\overset{t}{\underset{1e}{y}}\text{=}\overset{t}{\underset{\text{1}}{y}}-\overset{*}{\underset{\text{1}}{y}}$, $\overset{t}{\underset{2e}{Y}}\text{=}\overset{t}{\underset{\text{2}}{Y}}-\overset{*}{\underset{\text{2}}{Y}}$), then we obtain the following formula (For the detailed derivation process, please refer to the appendix C.):


$$\begin{array}{c} \begin{array}{c} \langle \overset{t}{\underset{2e}{Y}},FDiag\left(\overset{t+1}{\underset{e}{x}}\right)\rangle -\mu \langle -\overset{t}{\underset{e}{J}}+FDiag\left(\overset{t+1}{\underset{e}{x}}\right),FDiag\left(\overset{t+1}{\underset{e}{x}}\right)\rangle \\ -\langle \overset{t}{\underset{1e}{y}},WF\overset{t+1}{\underset{e}{x}}\rangle -\mu \langle \overset{t}{\underset{e}{\hat{e}}}+WF\overset{t+1}{\underset{e}{x}},WF\overset{t+1}{\underset{e}{x}}\rangle \geq 0 \end{array} \end{array}$$ (45)

Similarly, we have


$$\begin{array}{c} \langle \overset{t}{\underset{2e}{Y}},-\overset{t\text{+}1}{\underset{e}{J}}\rangle \text{+}\mu \langle FDiag\left(\overset{t+1}{\underset{e}{x}}\right)-\overset{t\text{+}1}{\underset{e}{J}},\overset{t\text{+}1}{\underset{e}{J}}\rangle \geq 0, \end{array}$$ (46)

$$\begin{array}{c} \langle \overset{t}{\underset{1e}{y}},-\overset{t\text{+}1}{\underset{e}{\hat{e}}}\rangle -\mu \langle \overset{t\text{+}1}{\underset{e}{\hat{e}}}+WF\overset{t+1}{\underset{e}{x}},\overset{t\text{+}1}{\underset{e}{\hat{e}}}\rangle \geq 0, \end{array}$$ (47)

Summing (45)-(47) together follows that


$$\begin{array}{c} \begin{array}{c} -\langle \overset{t}{\underset{1e}{y}},\overset{t\text{+}1}{\underset{e}{\hat{e}}}+WF\overset{t+1}{\underset{e}{x}}\rangle -\langle \overset{t}{\underset{2e}{Y}},\overset{t\text{+}1}{\underset{e}{J}}-FDiag\left(\overset{t+1}{\underset{e}{x}}\right)\rangle \\ \geq \mu \overset{\text{2}}{\underset{\text{2}}{\|WF\overset{t+1}{\underset{e}{x}}+\overset{t\text{+}1}{\underset{e}{\hat{e}}}\|}}+\mu \langle WF\overset{t+1}{\underset{e}{x}},\overset{t}{\underset{e}{\hat{e}}}-\overset{t\text{+}1}{\underset{e}{\hat{e}}}\rangle \\ +\mu \overset{\text{2}}{\underset{F}{\|FDiag\left(\overset{t+1}{\underset{e}{x}}\right)-\overset{t+1}{\underset{e}{J}}\|}}+\mu \langle FDiag\left(\overset{t+1}{\underset{e}{x}}\right),\overset{t\text{+}1}{\underset{e}{J}}-\overset{t}{\underset{e}{J}}\rangle \end{array} \end{array}$$ (48)

(The detailed derivation process can be found in Appendix D.)

Substituting (40) into (48), we obtain


$$\begin{array}{c} \begin{array}{c} \frac{\text{1}}{2\mu }\left(\overset{\text{2}}{\underset{\text{2}}{\|\overset{t}{\underset{1e}{y}}\|}}-\overset{\text{2}}{\underset{\text{2}}{\|\overset{t+1}{\underset{1e}{y}}\|}}\right)\text{+}\frac{\mu }{\text{2}}\overset{\text{2}}{\underset{\text{2}}{\|WF\overset{t+1}{\underset{e}{x}}+\overset{t\text{+}1}{\underset{e}{\hat{e}}}\|}}\text{+}\frac{\text{1}}{2\mu }\left(\overset{\text{2}}{\underset{F}{\|\overset{t}{\underset{2e}{Y}}\|}}-\overset{\text{2}}{\underset{F}{\|\overset{t+1}{\underset{2e}{Y}}\|}}\right)\\ \text{+}\frac{\mu }{\text{2}}\overset{\text{2}}{\underset{F}{\|\overset{t+1}{\underset{e}{J}}-FDiag\left(\overset{t+1}{\underset{e}{x}}\right)\|}}\geq \mu \overset{\text{2}}{\underset{\text{2}}{\|WF\overset{t+1}{\underset{e}{x}}+\overset{t\text{+}1}{\underset{e}{\hat{e}}}\|}}\text{+}\mu \langle WF\overset{t+1}{\underset{e}{x}},\overset{t}{\underset{e}{\hat{e}}}-\overset{t\text{+}1}{\underset{e}{\hat{e}}}\rangle \\ \text{+}\mu \overset{\text{2}}{\underset{F}{\|FDiag\left(\overset{t+1}{\underset{e}{x}}\right)-\overset{t+1}{\underset{e}{J}}\|}}+\mu \langle FDiag\left(\overset{t+1}{\underset{e}{x}}\right),\overset{t\text{+}1}{\underset{e}{J}}-\overset{t}{\underset{e}{J}}\rangle \end{array} \end{array}$$ (49)

With the simple transform of (49), we then have


$$\begin{array}{c} \begin{array}{c} \frac{\text{1}}{\mu }\left(\overset{\text{2}}{\underset{\text{2}}{\|\overset{t}{\underset{1e}{y}}\|}}-\overset{\text{2}}{\underset{\text{2}}{\|\overset{t+1}{\underset{1e}{y}}\|}}\right)\text{+}\frac{\text{1}}{\mu }\left(\overset{\text{2}}{\underset{F}{\|\overset{t}{\underset{2e}{Y}}\|}}-\overset{\text{2}}{\underset{F}{\|\overset{t+1}{\underset{2e}{Y}}\|}}\right)\geq \mu \overset{\text{2}}{\underset{\text{2}}{\|WF\overset{t+1}{\underset{e}{x}}+\overset{t\text{+}1}{\underset{e}{\hat{e}}}\|}}\\ \text{+2}\mu \langle WF\overset{t+1}{\underset{e}{x}},\overset{t}{\underset{e}{\hat{e}}}-\overset{t\text{+}1}{\underset{e}{\hat{e}}}\rangle \text{+}\mu \overset{\text{2}}{\underset{F}{\|FDiag\left(\overset{t+1}{\underset{e}{x}}\right)-\overset{t+1}{\underset{e}{J}}\|}}\\ +2\mu \langle FDiag\left(\overset{t+1}{\underset{e}{x}}\right),\overset{t\text{+}1}{\underset{e}{J}}-\overset{t}{\underset{e}{J}}\rangle \end{array} \end{array}$$ (50)

For the inequation (41), we have


$$\begin{array}{c} \lambda \left({\|J\|}_{*}-{\|J^{t\text{+1}}\|}_{*}\right)+\langle \overset{t}{\underset{\text{2}}{Y}},J-J^{t+1}\rangle +\mu \langle J^{t+1}-FDiag\left(x^{t+1}\right),J-J^{t+1}\rangle \geq 0 \end{array}$$ (51)

$$\begin{array}{c} \lambda \left({\|J\|}_{*}-{\|J^{t}\|}_{*}\right)+\langle \overset{t\text{-}1}{\underset{\text{2}}{Y}},J-J^{t}\rangle +\mu \langle J^{t}-FDiag\left(x^{t}\right),J-J^{t}\rangle \geq 0 \end{array}$$ (52)

By taking $J\text{=}J^{t}$ in (51) and $J\text{=}J^{t+1}$ in (52) respectively, and we add them together and take the aforementioned relative error into full account ($\overset{t}{\underset{e}{J}}\text{=}J^{t}-J^{*}$, $\overset{t}{\underset{e}{x}}\text{=}x^{t}-x^{*}$, $\overset{t}{\underset{e}{\hat{e}}}\text{=}{\hat{e}}^{t}-{\hat{e}}^{*}$, $\overset{t}{\underset{1e}{y}}\text{=}\overset{t}{\underset{\text{1}}{y}}-\overset{*}{\underset{\text{1}}{y}}$, $\overset{t}{\underset{2e}{Y}}\text{=}\overset{t}{\underset{\text{2}}{Y}}-\overset{*}{\underset{\text{2}}{Y}}$), then we obtain the following formula (For the detailed derivation process, please refer to the appendix E.):


$$\begin{array}{c} \langle \overset{t-1}{\underset{2e}{Y}}\text{-}\overset{t}{\underset{2e}{Y}},\overset{t+1}{\underset{e}{J}}-\overset{t}{\underset{e}{J}}\rangle -\mu \overset{\text{2}}{\underset{F}{\|\overset{t+1}{\underset{e}{J}}-\overset{t}{\underset{e}{J}}\|}}-\mu \langle FDiag\left(\overset{t}{\underset{e}{x}}-\overset{t+1}{\underset{e}{x}}\right),\overset{t+1}{\underset{e}{J}}-\overset{t}{\underset{e}{J}}\rangle \geq 0 \end{array}$$ (53)

In addition, using the relationship $\overset{t}{\underset{2e}{Y}}=\overset{t\text{-}1}{\underset{2e}{Y}}+\mu \left(\overset{t}{\underset{e}{J}}-FDiag\left(\overset{t}{\underset{e}{x}}\right)\right)$ to (53) leads to


$$\begin{array}{c} \overset{\text{2}}{\underset{F}{\|\overset{t+1}{\underset{e}{J}}-\overset{t}{\underset{e}{J}}\|}}\leq \langle FDiag\left(\overset{t+1}{\underset{e}{x}}\right)-\overset{t}{\underset{e}{J}},\overset{t+1}{\underset{e}{J}}-\overset{t}{\underset{e}{J}}\rangle , \end{array}$$ (54)

By the application of


$$\begin{array}{c} \langle \overset{t+1}{\underset{e}{J}}-\overset{t}{\underset{e}{J}},\overset{t}{\underset{e}{J}}\rangle \text{=}\frac{\text{1}}{\text{2}}\left(\overset{\text{2}}{\underset{F}{\|\overset{t+1}{\underset{e}{J}}\|}}-\overset{\text{2}}{\underset{F}{\|\overset{t}{\underset{e}{J}}\|}}-\overset{\text{2}}{\underset{F}{\|\overset{t+1}{\underset{e}{J}}-\overset{t}{\underset{e}{J}}\|}}\right), \end{array}$$ (55)

the inequation (54) can be rewritten as


$$\begin{array}{c} 2\langle FDiag\left(\overset{t+1}{\underset{e}{x}}\right),\overset{t+1}{\underset{e}{J}}-\overset{t}{\underset{e}{J}}\rangle \geq \overset{\text{2}}{\underset{F}{\|\overset{t+1}{\underset{e}{J}}\|}}-\overset{\text{2}}{\underset{F}{\|\overset{t}{\underset{e}{J}}\|}}\text{+}\overset{\text{2}}{\underset{F}{\|\overset{t+1}{\underset{e}{J}}-\overset{t}{\underset{e}{J}}\|}}, \end{array}$$ (56)

Similarly, we obtain


$$\begin{array}{c} 2\langle WF\overset{t+1}{\underset{e}{x}},\overset{t}{\underset{e}{\hat{e}}}-\overset{t\text{+}1}{\underset{e}{\hat{e}}}\rangle \geq \overset{\text{2}}{\underset{\text{2}}{\|\overset{t+1}{\underset{e}{\hat{e}}}\|}}-\overset{\text{2}}{\underset{\text{2}}{\|\overset{t}{\underset{e}{\hat{e}}}\|}}\text{+}\overset{\text{2}}{\underset{\text{2}}{\|\overset{t}{\underset{e}{\hat{e}}}-\overset{t\text{+}1}{\underset{e}{\hat{e}}}\|}}, \end{array}$$ (57)

Substituting (56) and (57) into (50), we have


$$\begin{array}{c} \begin{array}{c} \frac{\text{1}}{\mu }\left(\overset{\text{2}}{\underset{\text{2}}{\|\overset{t}{\underset{1e}{y}}\|}}-\overset{\text{2}}{\underset{\text{2}}{\|\overset{t+1}{\underset{1e}{y}}\|}}\right)\text{+}\frac{\text{1}}{\mu }\left(\overset{\text{2}}{\underset{F}{\|\overset{t}{\underset{2e}{Y}}\|}}-\overset{\text{2}}{\underset{F}{\|\overset{t+1}{\underset{2e}{Y}}\|}}\right)\geq \mu \overset{\text{2}}{\underset{\text{2}}{\|WF\overset{t+1}{\underset{e}{x}}+\overset{t\text{+}1}{\underset{e}{\hat{e}}}\|}}\\ \text{+}\mu \left(\overset{\text{2}}{\underset{\text{2}}{\|\overset{t+1}{\underset{e}{\hat{e}}}\|}}-\overset{\text{2}}{\underset{\text{2}}{\|\overset{t}{\underset{e}{\hat{e}}}\|}}\text{+}\overset{\text{2}}{\underset{\text{2}}{\|\overset{t}{\underset{e}{\hat{e}}}-\overset{t\text{+}1}{\underset{e}{\hat{e}}}\|}}\right)\text{+}\mu \overset{\text{2}}{\underset{F}{\|FDiag\left(\overset{t+1}{\underset{e}{x}}\right)-\overset{t+1}{\underset{e}{J}}\|}}\\ +\mu \left(\overset{\text{2}}{\underset{F}{\|\overset{t+1}{\underset{e}{J}}\|}}-\overset{\text{2}}{\underset{F}{\|\overset{t}{\underset{e}{J}}\|}}\text{+}\overset{\text{2}}{\underset{F}{\|\overset{t+1}{\underset{e}{J}}-\overset{t}{\underset{e}{J}}\|}}\right) \end{array} \end{array}$$ (58)

Then we get


$$\begin{array}{c} \begin{array}{c} \frac{\text{1}}{\mu }\left(\overset{\text{2}}{\underset{\text{2}}{\|\overset{t}{\underset{1e}{y}}\|}}-\overset{\text{2}}{\underset{\text{2}}{\|\overset{t+1}{\underset{1e}{y}}\|}}\right)\text{+}\frac{\text{1}}{\mu }\left(\overset{\text{2}}{\underset{F}{\|\overset{t}{\underset{2e}{Y}}\|}}-\overset{\text{2}}{\underset{F}{\|\overset{t+1}{\underset{2e}{Y}}\|}}\right)\text{+}\mu \left(\overset{\text{2}}{\underset{\text{2}}{\|\overset{t}{\underset{e}{\hat{e}}}\|}}-\overset{\text{2}}{\underset{\text{2}}{\|\overset{t\text{+}1}{\underset{e}{\hat{e}}}\|}}\right)\\ +\mu \left(\overset{\text{2}}{\underset{F}{\|\overset{t}{\underset{e}{J}}\|}}-\overset{\text{2}}{\underset{F}{\|\overset{t\text{+}1}{\underset{e}{J}}\|}}\right)\geq \mu \overset{\text{2}}{\underset{\text{2}}{\|WF\overset{t+1}{\underset{e}{x}}+\overset{t\text{+}1}{\underset{e}{\hat{e}}}\|}}\text{+}\mu \overset{\text{2}}{\underset{\text{2}}{\|\overset{t}{\underset{e}{\hat{e}}}-\overset{t\text{+}1}{\underset{e}{\hat{e}}}\|}}\\ \text{+}\mu \overset{\text{2}}{\underset{F}{\|FDiag\left(\overset{t+1}{\underset{e}{x}}\right)-\overset{t+1}{\underset{e}{J}}\|}}+\mu \overset{\text{2}}{\underset{F}{\|\overset{t+1}{\underset{e}{J}}-\overset{t}{\underset{e}{J}}\|}} \end{array} \end{array}$$ (59)

By summing the inequality (59) from t=0 to t=N, we obtain


$$\begin{array}{c} \begin{array}{c} \frac{\text{1}}{\mu }\left(\overset{\text{2}}{\underset{\text{2}}{\|\overset{\text{0}}{\underset{1e}{y}}\|}}-\overset{\text{2}}{\underset{\text{2}}{\|\overset{N+1}{\underset{1e}{y}}\|}}\right)\text{+}\frac{\text{1}}{\mu }\left(\overset{\text{2}}{\underset{F}{\|\overset{\text{0}}{\underset{2e}{Y}}\|}}-\overset{\text{2}}{\underset{F}{\|\overset{N+1}{\underset{2e}{Y}}\|}}\right)\text{+}\mu \left(\overset{\text{2}}{\underset{\text{2}}{\|\overset{\text{0}}{\underset{e}{\hat{e}}}\|}}-\overset{\text{2}}{\underset{\text{2}}{\|\overset{N\text{+}1}{\underset{e}{\hat{e}}}\|}}\right)\\ +\mu \left(\overset{\text{2}}{\underset{F}{\|\overset{\text{0}}{\underset{e}{J}}\|}}-\overset{\text{2}}{\underset{F}{\|\overset{N\text{+}1}{\underset{e}{J}}\|}}\right)\geq \mu \sum_{t=0}^{N}\overset{\text{2}}{\underset{\text{2}}{\|WF\overset{t+1}{\underset{e}{x}}+\overset{t\text{+}1}{\underset{e}{\hat{e}}}\|}}\text{+}\mu \sum_{t=0}^{N}\overset{\text{2}}{\underset{\text{2}}{\|\overset{t}{\underset{e}{\hat{e}}}-\overset{t\text{+}1}{\underset{e}{\hat{e}}}\|}}\\ \text{+}\mu \sum_{t=0}^{N}\overset{\text{2}}{\underset{F}{\|FDiag\left(\overset{t+1}{\underset{e}{x}}\right)-\overset{t+1}{\underset{e}{J}}\|}}+\mu \sum_{t=0}^{N}\overset{\text{2}}{\underset{F}{\|\overset{t+1}{\underset{e}{J}}-\overset{t}{\underset{e}{J}}\|}} \end{array} \end{array}$$ (60)

With the simple transform of (60), we then have


$$\begin{array}{c} \begin{array}{c} \frac{\text{1}}{\mu }\overset{\text{2}}{\underset{\text{2}}{\|\overset{\text{0}}{\underset{1e}{y}}\|}}\text{+}\frac{\text{1}}{\mu }\overset{\text{2}}{\underset{F}{\|\overset{\text{0}}{\underset{2e}{Y}}\|}}\text{+}\mu \overset{\text{2}}{\underset{\text{2}}{\|\overset{\text{0}}{\underset{e}{\hat{e}}}\|}}+\mu \overset{\text{2}}{\underset{F}{\|\overset{\text{0}}{\underset{e}{J}}\|}}\geq \mu \sum_{t=0}^{N}\overset{\text{2}}{\underset{\text{2}}{\|WF\overset{t+1}{\underset{e}{x}}+\overset{t\text{+}1}{\underset{e}{\hat{e}}}\|}}\\ \text{+}\mu \sum_{t=0}^{N}\overset{\text{2}}{\underset{\text{2}}{\|\overset{t}{\underset{e}{\hat{e}}}-\overset{t\text{+}1}{\underset{e}{\hat{e}}}\|}}\text{+}\mu \sum_{t=0}^{N}\overset{\text{2}}{\underset{F}{\|FDiag\left(\overset{t+1}{\underset{e}{x}}\right)-\overset{t+1}{\underset{e}{J}}\|}}+\mu \sum_{t=0}^{N}\overset{\text{2}}{\underset{F}{\|\overset{t+1}{\underset{e}{J}}-\overset{t}{\underset{e}{J}}\|}} \end{array} \end{array}$$ (61)

which implies that


$$\begin{array}{c} \underset{t\to \infty }{lim}\overset{\text{2}}{\underset{\text{2}}{\|WF\overset{t+1}{\underset{e}{x}}+\overset{t\text{+}1}{\underset{e}{\hat{e}}}\|}}=0,\text{\textbackslash{}hspace\{0.17em}\}\underset{t\to \infty }{lim}\overset{\text{2}}{\underset{\text{2}}{\|\overset{t}{\underset{e}{\hat{e}}}-\overset{t\text{+}1}{\underset{e}{\hat{e}}}\|}}=0, \end{array}$$

$$\begin{array}{c} \underset{t\to \infty }{lim}\overset{\text{2}}{\underset{F}{\|FDiag\left(\overset{t+1}{\underset{e}{x}}\right)-\overset{t+1}{\underset{e}{J}}\|}}=0,\text{\textbackslash{}hspace\{0.17em}\}\underset{t\to \infty }{lim}\overset{\text{2}}{\underset{F}{\|\overset{t+1}{\underset{e}{J}}-\overset{t}{\underset{e}{J}}\|}}=0, \end{array}$$

Then so there exists a convergent subsequence $\left\{\left(J^{t_{l}\text{+1}},x^{t_{l}\text{+1}},{\hat{e}}^{t_{l}+1}\right)\right\}$, i.e.,


$$\begin{array}{c} \underset{t\to \infty }{lim}\left\{\left(J^{t_{l}\text{+1}},x^{t_{l}\text{+1}},{\hat{e}}^{t_{l}+1}\right)\right\}\text{=}\left(J^{⋄},x^{⋄},{\hat{e}}^{⋄}\right). \end{array}$$

Now we need to show that $\left(J^{⋄},x^{⋄},{\hat{e}}^{⋄},\overset{⋄}{\underset{\text{1}}{y}},\overset{⋄}{\underset{\text{2}}{Y}}\right)$ is the saddle point of the Lagrangian function. In fact, using the iteration scheme (25)-(29) we can deduce that

$\left\{ \begin{array}{c} @c@{\hat{e}}^{⋄}\text{=}W\left(y-Fx^{⋄}\right)\\ J^{⋄}=FDiag\left(x^{⋄}\right) \end{array}\right.\text{\textbackslash{}hspace\{0.17em}\}and\text{\textbackslash{}hspace\{0.17em}\}\left\{ \begin{array}{c} @c@\underset{t\to \infty }{lim}\overset{t}{\underset{\text{1}}{y}}=\overset{⋄}{\underset{\text{1}}{y}}\\ \underset{t\to \infty }{lim}\overset{t}{\underset{\text{2}}{Y}}=\overset{⋄}{\underset{\text{2}}{Y}} \end{array}\right.,$

This implies that $\left(J^{⋄},x^{⋄},{\hat{e}}^{⋄},\overset{⋄}{\underset{\text{1}}{y}},\overset{⋄}{\underset{\text{2}}{Y}}\right)$ is the saddle point, that is to say,


$$\begin{array}{c} \left(J^{⋄},x^{⋄},{\hat{e}}^{⋄},\overset{⋄}{\underset{\text{1}}{y}},\overset{⋄}{\underset{\text{2}}{Y}}\right)\text{=}\left(J^{*},x^{*},{\hat{e}}^{*},\overset{*}{\underset{\text{1}}{y}},\overset{*}{\underset{\text{2}}{Y}}\right), \end{array}$$

Furthermore, it is easy to deduce that the sequence $\left\{\left(J^{t\text{+1}},x^{t\text{+1}},{\hat{e}}^{t+1},{y_{\text{1}}}^{t+\text{1}},{Y_{\text{2}}}^{t\text{+1}}\right)\right\}$ is convergent.

### Classification

Based on the above optimization results, we obtain the optimal solution of the model $x^{*}=\left[\overset{*}{\underset{p}{x}};\overset{*}{\underset{v}{x}}\right]$ and the corresponding weighted matrix $W^{*}$. For each class i, let $\delta_{i}:R^{n}\to R^{n}$ be the characteristic function that selects the coefficients associated with the i th class. For $\overset{*}{\underset{P}{x}}\in R^{n\times 1}$, $\delta_{i}\left(\overset{*}{\underset{P}{x}}\right)$ is a new vector whose only nonzero entries are the entries in $\overset{*}{\underset{P}{x}}$ that are associated with class i. Using only the coefficients associated with the i th class, one can approximate the given test sample y as $\hat{y}=\left[P,V\right]\left[\delta_{i}\left(\overset{*}{\underset{p}{x}}\right);\overset{*}{\underset{v}{x}}\right]$. Since random error usually leads to a larger reconstruction error than that of the ordinary image, this part increases the weight function to detect large random errors in the test sample and assigns a certain weight to make it output a smaller value, ultimately reducing the impact of random error in the image recognition process. We then give the residual between y and $\hat{y}$ as follows:


$$\begin{array}{c} {\text{r}}_{i}\left(y\right)={\|W^{*}\left(y-\left[P,V\right]\left[\begin{array}{c} @c@\delta_{i}\left(\overset{*}{\underset{p}{x}}\right)\\ \overset{*}{\underset{v}{x}} \end{array}\right]\right)\|}_{\text{2}},i=1,2,\text{\textbackslash{}ldots},k \end{array}$$ (62)

Thus, based on the above residual, the test samples y are assigned to the class that minimizes the refactored residuals that is $Identity\left(y\right)=arg\underset{i}{min}r_{i}\left(y\right)$. Based on the above analysis, this algorithm can be summarized into algorithm 2:

**Algorithm 2:**

Input: the dictionary $A\in R^{m\times n}$, the test image $y\in R^{m}$

Output: the label of test samples y

(1) Compute the fusion dictionary $F=\left[P,V\right]\in R^{m\times \left(k+n\right)}$ via (18);

(2) Solve the minimization problem as follow according to algorithm 1;

$min{\|W\left(y-\left[P,V\right]\left[\begin{array}{c} @c@x_{p}\\ x_{v} \end{array}\right]\right)\|}_{\text{1}}+\lambda {\|\left[P,V\right]Diag\left[\begin{array}{c} @c@x_{p}\\ x_{v} \end{array}\right]\|}_{*}$

(3) Compute the residuals

${\text{r}}_{i}\left(y\right)={\|W^{*}\left(y-\left[P,V\right]\left[\begin{array}{c} @c@\delta_{i}\left(\overset{*}{\underset{p}{x}}\right)\\ \overset{*}{\underset{v}{x}} \end{array}\right]\right)\|}_{\text{2}},i=1,2,\text{\textbackslash{}ldots},k$

(4) Predict the identity of y:

$label\left(y\right)=arg\underset{i}{min}r_{i}\left(y\right)$

### Flow Diagram

**Fig 1:**
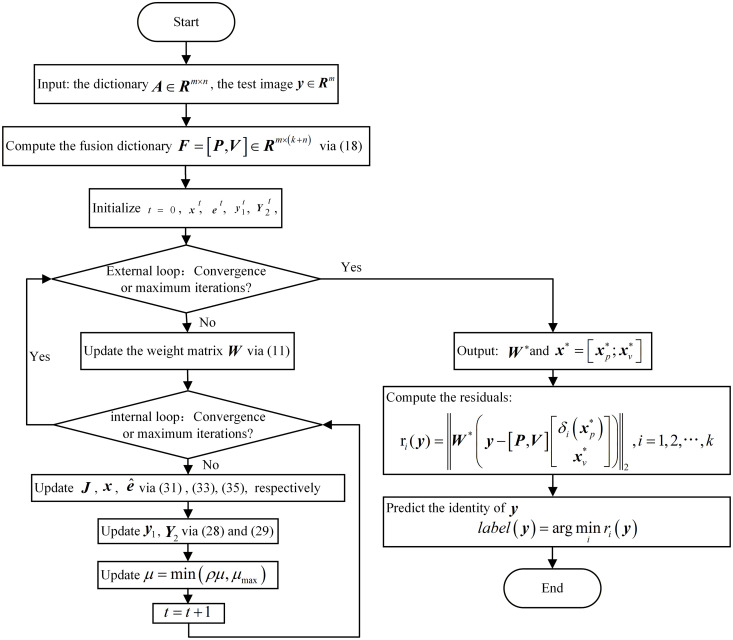
Flowchart illustrating the overall pipeline of the proposed ASRC-WFD method.

Fig 1 shows the flow diagram of the proposed ASRC-WFD. As can be seen from Fig 1, the adaptive robust sparse representation algorithm based on weighted and fusion dictionary is mainly divided into three stages: fusion dictionary construction, weight function update, and sparse coefficient optimization. This study focuses on face recognition under insufficient sampling scenarios. The constructed fusion dictionary can enhance the completeness of dictionary representation in the case of limited training samples. On the basis of the fusion dictionary, the introduced weighted $l_{\text{1}}$ -norm constraint can effectively suppress complex error interference in face images. In addition, the nuclear norm constraint imposed on sparse representation coefficients can reasonably balance the sparsity of representation and the correlation information of image data. It can be observed from the flowchart that the proposed algorithm involves iterative calculation, which is mainly reflected in the solving process of weight function and sparse coefficients. The iterative optimization of weight parameters and sparse coefficients enables the presented adaptive robust sparse representation algorithm with weighted and fusion dictionary to converge to the optimal solution.

## Results

To evaluate the efficacy of our proposed adaptive sparse representation model with weighted and fusion dictionaries, we conducted comprehensive experiments on several widely-used face recognition benchmarks, including the (http://RVL.www.ecn.purdue.edu) AR [33], (https://www.cl.cam.ac.uk/research/dtg/attarchive/facedatabase.html) ORL [34] (http://cvc.yale.edu/projects/yalefaces/yalefaces.html) Yale [35], and (http://www.ri.cmu.edu/projects/project_418.html) CMU PIE [36]. A comparative analysis was performed between the proposed method and a range of established face recognition algorithms, such as NN[1], LRC [6], SRC [10], CRC [8], SLRC [12], ASRC [10] and RSC [13]. For feature extraction, we employed Principal Component Analysis (PCA) on all face image datasets.

The setting of parameters b and c in (4) follows the recommendations of literature [22].They are estimated in each iteration using the mean em of the absolute value of coding residuals. Specifically, b and c are set to $\overset{t}{\underset{m}{e}}/k$ and $c_{\text{0}}exp\left(-\overset{t}{\underset{m}{e}}/b\right)$, respectively. In the experiment, we set $c_{\text{0}}=0.001$ and k=0.5, μ in (17) is set to ${2m/\|y\|}_{\text{1}}$.The iteration number of the inner loop and the outer loop in Algorithm 1 are set to 10 and 30, respectively.

### Face recognition with clean face

This part verifies the basic recognition performance of all methods with clean face image recognition.

### AR database

The AR database comprises over 4,000 frontal face images of 126 subjects. In this experiment, a subset of 50 male and 50 female subjects was selected. Each subject provided 14 frontal images with only illumination and expression variations. All images were cropped to a size of 120 × 165 pixels. To evaluate performance under different training conditions, two experimental scenarios were constructed: one with limited data (2 images per subject for training and the rest for testing) and one with ample data (6 images per subject for training, the rest for testing). Feature dimensions were varied across experiments, the corresponding results summarized in Table 1 and Table 2.

**Table 1:** Recognition rates (%) based on 2 images per subject for training on the AR database.

Dimension of feature space	NN	LRC	SRC	CRC	SLRC	ASRC	RSC	ASRC-WFD
**30**	61.00	63.08	62.58	51.75	62.50	55.58	**63.67**	63.41
**60**	67.17	69.58	69.58	66.67	72.17	68.33	71.67	**72.26**
**90**	68.58	71.33	76.08	75.17	76.50	75.67	76.42	**78.36**
**120**	69.75	72.75	77.92	76.83	78.25	77.42	78.08	**79.01**
**150**	69.83	73.42	78.75	79.33	79.50	78.67	78.83	**80.77**
**180**	70.17	73.83	79.00	79.75	80.58	79.67	79.00	**80.77**

**Table 2:** Recognition rates (%) based on 6 images per subject for training on the AR database.

Dimension of feature space	NN	LRC	SRC	CRC	SLRC	ASRC	RSC	ASRC-WFD
**30**	50.50	46.88	50.25	39.63	44.00	43.25	**54.50**	52.01
**90**	60.38	62.38	72.75	65.50	68.38	71.88	73.25	**80.83**
**150**	62.38	66.25	76.50	75.25	77.25	77.75	75.38	**83.75**
**200**	62.62	67.38	77.50	76.38	79.25	79.75	75.63	**85.88**
**300**	62.25	68.00	78.63	78.63	82.13	80.50	76.50	**86.00**
**400**	62.00	68.13	79.00	81.63	83.63	82.25	76.50	**87.00**
**500**	62.75	68.63	78.88	84.63	85.00	82.50	76.50	**87.25**

As shown in Table 1, the proposed ASRC-WFD consistently outperforms all compared methods across nearly all experimental settings. The performance advantage is most pronounced under sufficient training samples and higher-dimensional feature spaces, achieving a recognition rate of 87.25% with 6 training samples per subject at 500 feature dimensions. This gain is attributed to the integrated modeling of sparsity, correlation, and robustness. Even with limited training data, the fusion dictionary and nuclear norm constraints effectively capture intra-class variations.

SLRC generally surpasses SRC due to its collaborative robustness under sparse sampling. ASRC excels in sufficient-data regimes but underperforms under limited samples, as it overlooks dictionary incompleteness when leveraging sample correlations. While SRC, CRC, SLRC, ASRC, and RSC achieve competitive results, all are consistently outperformed by ASRC-WFD. NN performs the weakest due to its sensitivity to facial similarity, and LRC, though stronger, remains inferior to our method.

In summary, the proposed ASRC-WFD demonstrates superior recognition performance under noiseless conditions on the AR database.

### ORL database

The ORL database contains faces of 40 subjects with diverse ages, genders, and racial backgrounds. Image variations include changes in illumination, pose, facial accessories, and expressions. To evaluate robustness under limited data, we constructed two training scenarios: 2 images per subject (insufficient) and 4 images per subject (sufficient), with the remaining images used for testing. Recognition performance across varying feature dimensions is reported in Tables 3 and 4.

**Table 3:** Recognition rates (%) based on 2 images per subject for training on the ORL database.

Dimension of feature space	NN	LRC	SRC	CRC	SLRC	ASRC	RSC	ASRC-WFD
**20**	73.75	72.19	80.31	70.63	77.81	72.50	81.25	**83.02**
**30**	76.88	75.63	84.06	79.06	85.94	78.75	83.75	**86.79**
**40**	78.13	76.25	84.69	80.00	86.88	83.75	84.38	**87.11**
**50**	77.50	76.88	84.06	81.25	87.50	85.31	84.38	**87.11**
**60**	77.19	77.50	85.00	81.56	86.56	84.38	85.00	**87.42**
**79**	77.81	77.81	86.25	82.81	87.50	85.00	86.56	**88.05**

**Table 4:** Recognition rates (%) based on 4 images per subject for training on the ORL database.

Dimension of feature space	NN	LRC	SRC	CRC	SLRC	ASRC	RSC	ASRC-WFD
**30**	82.92	85.00	89.17	83.33	87.92	82.92	89.58	**91.67**
**60**	83.33	83.75	90.00	88.75	91.25	87.92	90.00	**92.08**
**80**	84.17	83.75	90.00	89.58	91.67	88.75	89.58	**92.47**
**120**	84.17	85.00	91.25	90.42	91.67	90.00	90.83	**92.02**
**140**	84.58	84.58	91.25	90.42	**92.92**	89.17	90.83	92.02
**159**	83.75	84.17	91.25	90.42	**92.92**	89.58	90.42	92.02

As shown in Table 3, ASRC-WFD achieves the highest recognition rates across most settings, except at 50 dimensions. This advantage is attributed to the fusion dictionary, which enhances dictionary completeness, and the effective utilization of correlation information between images. Under insufficient training samples, ASRC-WFD reaches a maximum recognition rate of 88.05%, outperforming NN, LRC, SRC, CRC, SLRC, ASRC, and RSC by 10.24%, 10.24%, 1.80%, 5.24%, 0.55%, 3.05%, and 1.49%, respectively.

Results in Table 4 show that the highest recognition rates for most algorithms do not occur at the maximum feature dimension. For instance, with sufficient training samples, NN and RSC perform best at 140 dimensions, while LRC, SRC, CRC, and ASRC achieve optimal results at 120 dimensions. Similarly, the proposed ASRC-WFD attains its peak performance of 92.02% at 120 dimensions. Overall, ASRC-WFD consistently surpasses all compared methods under noiseless conditions on the ORL database.

### Yale database

The Yale database comprises 15 subjects, each with 11 images that capture varying facial expressions and illumination conditions. These images were collected under different emotional states (e.g., sad, happy, surprised). In the experiments, 2 and 4 images per subject were used for training to simulate insufficient and sufficient sample scenarios, respectively, with the remaining images reserved for testing. Recognition performance under varying feature dimensions is summarized in Tables 5 and 6.

**Table 5:** Recognition rates (%) based on 2 images per subject for training on the Yale database.

Dimension of feature space	NN	LRC	SRC	CRC	SLRC	ASRC	RSC	ASRC-WFD
**15**	78.52	81.48	82.96	83.70	80.00	85.19	82.96	**87.31**
**20**	80.74	82.96	85.19	84.44	85.93	85.19	82.22	**87.31**
**25**	81.48	83.70	85.19	86.67	85.93	86.67	82.96	**88.81**
**29**	83.70	83.70	85.15	85.93	87.41	86.67	82.22	**89.55**

**Table 6:** Recognition rates (%) based on 4 images per subject for training on the Yale database.

Dimension of feature space	NN	LRC	SRC	CRC	SLRC	ASRC	RSC	ASRC-WFD
**20**	82.86	83.81	83.81	85.71	81.91	83.81	83.81	**88.46**
**30**	81.90	84.76	83.81	85.71	80.95	84.72	84.76	**86.54**
**40**	80.00	84.76	83.81	86.67	85.71	86.67	84.76	**87.50**
**50**	82.86	85.71	85.71	86.67	86.67	86.67	85.71	**89.42**

From Tables 5 and 6, we can see that ASRC-WFD obtains the best recognition rates at all levels. The performances of all the methods improve as the dimension of the feature space increase, and the proposed ASRC-WFD always remains the best. For example, when the number of training samples is 2 and the dimension of feature space is 29, the best recognition rate for ASRC-WFD on the Yale database is 89.55%, compared to 83.70% for NN and LRC, 85.15% for SRC, 85.93% for CRC, 87.41% for SLRC, 86.67% for ASRC, 82.22% for RSC. Since RSC has certain limitations in the case of insufficient sampling, it does not show superior performance in the experiment with 2 training samples per subject. In the experiment with the number of training samples of 4, the training samples are relatively more sufficient, so the recognition rate of the RSC algorithm is improved.

### CMU PIE database

To evaluate the generalizability of our algorithm for pattern recognition tasks, we conducted experiments on the C07 subset of the CMU PIE database. This subset contains 1,629 images of 68 subjects, with variations in facial expression, pose, and illumination. All images were cropped to a resolution of 64 × 64 pixels. In our experiments, four images per subject were used for training, with the remainder constituting the test set. The corresponding results are presented in Table 7.

**Table 7:** Recognition rates (%) based on 4 images per subject for training on the Yale database.

Dimension of feature space	NN	LRC	SRC	CRC	SLRC	ASRC	RSC	ASRC-WFD
**90**	42.87	60.37	56.32	56.18	59.49	58.46	59.85	**62.52**
**120**	43.16	62.65	59.04	58.16	61.54	59.78	62.21	**64.80**
**150**	43.01	63.24	60.81	59.34	63.16	60.15	64.34	**67.30**
**180**	43.01	63.53	62.06	60.22	63.97	60.66	65.29	**68.70**
**210**	43.24	63.97	62.28	60.66	64.85	60.74	66.03	**70.47**
**240**	43.38	63.75	62.65	60.88	65.88	60.74	66.40	**71.28**
**270**	43.68	64.04	63.02	61.47	66.25	60.74	67.79	**72.39**

As shown in Table 7, the proposed ASRC-WFD consistently outperforms all com-pared methods across every feature dimension. Our approach demonstrates a clear ad-vantage over conventional sparse representation-based methods such as SRC. Since each sample in the database exhibits unique facial expressions, poses, and illumination conditions, the extended dictionary in SLRC effectively captures inter-sample varia-tions, leading to its superior performance over SRC at all dimensions. Similarly, ASRC (which leverages both sparsity and correlation) and RSC (with its weighted design) also achieve higher recognition rates than SRC across dimensional settings.

The proposed method attains the highest recognition accuracy by comprehensive-ly integrating a fusion dictionary, correlation structure, and an adaptive weighting function to handle complex error patterns in face imagery. In summary, these experi-mental results confirm that ASRC-WFD constitutes an effective and robust solution for general pattern recognition tasks.

### Face recognition with random noises

This section evaluates the robustness of the proposed method against random noise corruption. Test samples were contaminated with random noise ranging from 10% to 80% in intensity. All compared methods were assessed under noise conditions from 0% to 80%. Here, 10% random noise indicates that 10% of the pixels were replaced with random values at random locations.

### AR database

**Fig 2:**
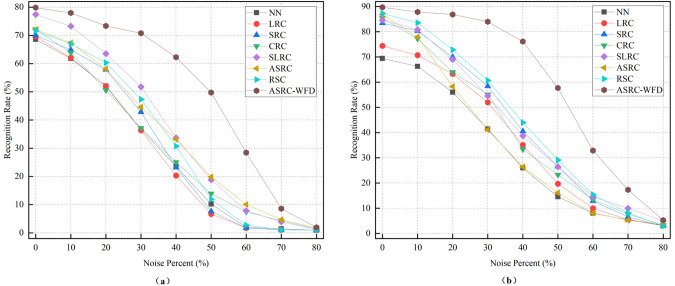
The recognition results of the AR database under random noise conditions. (a) The number of training samples per class was 2. (b) The number of training samples per class was 7.

For face recognition under random noise conditions, experiments were conducted from two perspectives on the AR database. Two evaluation settings were adopted: insufficient training samples and sufficient training samples. In the insufficient sample setting, two sample images per subject exhibiting only illumination and expression variations were selected from the 14 available images to form the training set, with the remaining images used for testing. Under the sufficient sample setting, half of the images per subject were used for training and the remainder for testing. All images were resized to 27 × 20 pixels, with experimental results presented in Fig 2.

As shown in Fig 2, the proposed ASRC-WFD method performs significantly better than the seven compared algorithms under random noise on the AR database.

In the insufficient-sample scenario (Fig 4(a)), as noise increases to 30%, our method declines gradually while others degrade sharply, demonstrating its stability under insufficient samples. Under sufficient samples (Fig 2(b)), our method maintains over 84% accuracy up to 30% noise, whereas NN, LRC, SRC, CRC, SLRC, ASRC, and RSC drop to 41.43%, 52.00%, 58.43%, 55.14%, 54.57%, 41.14%, and 60.71%, respectively. At 40% noise, our method achieves 76.41%, significantly exceeding RSC (44.00%), which benefits only from weighted norm constraints for outlier suppression. By integrating error modeling and correlation-guided recovery, our approach preserves performance even at 50% noise (57.71%), outperforming all others by at least 28.57%. These results validate the robustness of our method under both sampling conditions on AR.

**Fig 3:**
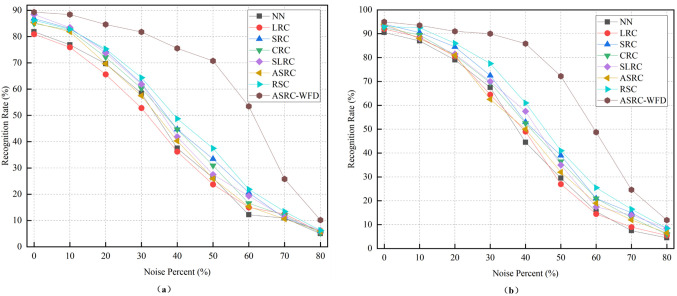
The recognition results of the ORL database under random noise condition. (a) The number of training samples per class was 2. (b) The number of training samples per class was 5.

### ORL database

The proposed method was evaluated on the ORL database under two protocols: one with insufficient training samples and the other with sufficient samples. In these experiments, 2 and 5 sample images per subject were randomly selected for training, respectively, with the remaining images used for testing. All images were resized to 16 × 16 pixels, and the results are presented in Fig 3.

Fig 3 demonstrates the superior performance and robustness of the proposed method against random noise on the ORL database, under both insufficient and sufficient training samples. Under insufficient samples (Fig 3(a)), our method maintains the highest recognition rate even at 60% noise (53.48%), owing to the fusion dictionary and weighted norm constraint, which enhance dictionary completeness and error tolerance. With sufficient samples (Fig 3(b)), our method also shows minimal performance degradation at 30% noise and significantly outperforms all seven benchmarks at 50% noise, with a recognition rate of 72.22%. This confirms the method’s exceptional robustness across different training conditions.

### Yale database

**Fig 4:**
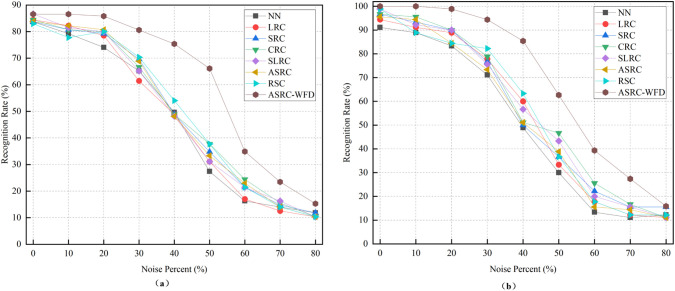
The recognition results of the Yale database under random noise condition. (a) The number of training samples per class was 2. (b) The number of training samples per class was 5.

The robustness of the proposed method was evaluated on the Yale database under both insufficient and sufficient training sample conditions with random noise. In the experiments, 2 and 5 images per subject were used for training, respectively, while the rest were reserved for testing. All images were resized to a resolution of 27 × 20 pixels. The corresponding results are shown in Figure 4.

Experimental results on the Yale database under insufficient sampling (Fig 4(a)) confirm the superior robustness of the proposed method. Its recognition rate remains almost unchanged with 20% random noise, outperforming all comparison methods, which show a clear decline. This stability is conferred by the synergistic action of the weight function and image correlation information. While the recognition rate of the proposed method drops from 85.82% to 66.15% as noise increases from 20% to 50%, it maintains a significant advantage; the comparison methods fall below 40%. This is explained by the progressive loss of discriminative facial features and inter-image correlation due to severe noise. As shown in Figure 4(b), the method’s robustness is also validated under sufficient sampling. Overall, the proposed method exhibits consistent and effective performance against random noise on the Yale database.

### CMU PIE database

**Fig 5:**
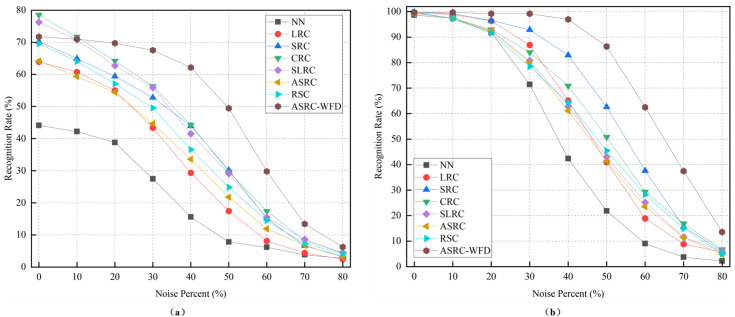
The recognition results of the CMU PIE database under random noise condition. (a) The number of training samples per class was 4; (b) The number of training samples per class was 10.

To evaluate the proposed method’s performance under varying sampling conditions (simulated by training with 4 and 10 samples per subject) and noise levels (from 10% to 80%), we conducted experiments on the CMU PIE sub-database C07, with the results shown in Fig 5.

Experimental results on the CMU PIE database under insufficient sampling (Fig 5(a)) reveal that the proposed method, while initially inferior to CRC and SLRC in a noiseless setting, exhibits superior noise tolerance. A sharp performance degradation is observed in CRC, SLRC, and RSC at 30% noise, in contrast to the gradual decline of the proposed method. This gradual degradation and sustained superior performance are due to the enhanced dictionary completeness and the introduction of a weight function that mitigates the impact of errors.

Under sufficient sampling (Figure 5(b)), the proposed method maintains a recognition rate of 99.16% at 30% noise, showing no performance loss, and significantly outperforms the best comparator (SRC at 92.86%). Its advantage is further amplified at higher noise levels, which is attributed to the integrated contribution of the fusion dictionary, image correlation, and error modeling. Overall, the method confirms strong robustness against random noise on the CMU PIE database.

### Face recognition with random occlusions

This section assesses the method’s robustness to random occlusion – a common real-world challenge where the position and size of obstructions are unpredictable. We test on multiple databases by occluding each test image with black blocks at random, unknown locations.

### AR database

Under the insufficient and sufficient sampling protocols, 2 and 7 samples per subject from the AR database were used for training, respectively, while the remainder were occluded with random black blocks for testing, the quantitative results are recorded in Table 8.

**Table 8:** Recognition rates (%) based on 2 images per subject for training on the AR database.

Number of training samples	NN	LRC	SRC	CRC	SLRC	ASRC	RSC	ASRC-WFD
**2**	63.17	64.33	65.67	65.25	66.50	65.17	67.33	**68.11**
**7**	60.00	63.29	68.14	67.57	69.00	70.86	70.71	**71.29**

The performance evaluation on the AR database under random occlusion reveals distinct behaviors among the comparison methods, which contextualizes the superiority of our approach. Sensitivity to occlusion explains the poor performance of NN and the instability of LRC. While SRC, CRC, SLRC, and ASRC achieve higher rates through strategies like sparsity or collaborative representation, they lack a dedicated mechanism for handling severe, random corruption. RSC, though robust in other error scenarios, does not excel here. In contrast, our method explicitly addresses the core challenge: it successfully characterizes random occlusions by integrating a weight constraint to suppress errors and leveraging correlation information to preserve essential features. This targeted design is why our method maintains the best stability against random occlusion.

### ORL database

In this experiment on the ORL database, 2 and 5 samples per subject were used for training under the insufficient and sufficient sampling conditions, respectively. The remaining samples were used for testing, where they were occluded with black blocks at random locations. The experimental results are presented in Table 9.

**Table 9:** The recognition rates (%) on the ORL database under occlusion.

Number of training samples	NN	LRC	SRC	CRC	SLRC	ASRC	RSC	ASRC-WFD
**2**	74.38	72.19	74.69	74.69	75.31	75.63	75.00	**76.73**
**5**	81.50	83.00	85.00	84.00	82.00	83.00	84.00	**87.44**

As evidenced in Table 9, our method demonstrates robust performance against random occlusion under both sampling conditions, yielding the highest recognition rates. This advantage stems from the synergistic effect of the fusion dictionary and image correlation, coupled with a weighted norm constraint that effectively suppress-es random errors.

### Yale database

This experiment evaluates the performance of the proposed algorithm on the Yale database when face images are occluded by random black blocks. For this purpose, 2 and 5 samples per subject were used for training under insufficient and sufficient conditions, respectively, while the remaining samples were used for testing. The experimental re-sults are presented in Table 10.

**Table 10:** The recognition rates (%) on the Yale database under occlusion.

Number of training samples	NN	LRC	SRC	CRC	SLRC	ASRC	RSC	ASRC-WFD
**2**	78.52	81.48	82.96	82.22	85.19	83.70	85.19	**86.57**
**5**	91.11	93.33	94.44	95.56	96.67	95.56	96.67	**98.88**

The experimental results in Table 10 confirm that the proposed method secures the highest recognition rate on the Yale database, regardless of sampling sufficiency. This performance is owing to its incorporation of a weighted $l_{\text{1}}$ norm constraint, which is critical for suppressing error propagation during recognition. The introduction of random block masks, which create pronounced localized errors, precisely create the conditions under which this robustness is most convincingly demonstrated.

### CMU PIE database

The algorithm’s performance under random block occlusion was further verified on the CMU PIE database using 4 training samples per subject. The test set was occluded with random black blocks, and the results are summarized in Table 11.

**Table 11:** The recognition rates (%) on the CMU PIE database under occlusion.

Number of training samples	NN	LRC	SRC	CRC	SLRC	ASRC	RSC	ASRC-WFD
**4**	91.54	93.75	94.85	95.22	95.96	95.59	95.59	**96.32**

As shown in Table 11, the proposed method achieves the highest recognition rate on the CMU PIE database under random occlusion. Compared with NN, LRC, SRC, CRC, SLRC, ASRC, and RSC, it achieves performance improvements of 4.78, 2.57, 1.47, 1.10, 0.36, 0.73, and 0.73 percentage points, respectively. This superiority stems from the method’s integration of sparsity and correlation information, coupled with the use of a weighted norm to suppress the influence of errors on the recognition results. Therefore, the proposed method also demonstrates strong robustness for occluded face recognition on the CMU PIE database.

### Ablation study

To quantitatively verify the effectiveness and necessity of each core component in the proposed algorithm, we conduct comprehensive ablation experiments on the ORL database under clean faces, random noise and random occlusion conditions. As shown in Table 12, the traditional baseline methods (NN, LRC, SRC, CRC) show limited robustness, and their recognition accuracy drops sharply under noise interference.

**Table 12:** Ablation results (%) of different modules of the proposed method.

Methods	Clean Face	Random Noise	Random Occlusion
**NN**	84.17	67.50	81.50
**LRC**	83.75	64.50	83.00
**SRC**	90.00	72.50	85.00
**CRC**	89.58	69.50	84.00
**Ours (Nuclear Norm)**	87.39	86.36	81.54
**Ours (Nuclear Norm** **+ Weighted Constraint)**	87.39	86.36	82.05
**Ours (Nuclear Norm +** **Weighted + Fusion Dictionary)**	**92.47**	**90.00**	**87.44**

Based on the internal module ablation results, the following conclusions can be drawn:

(1) The single nuclear norm constraint achieves promising noise robustness, but it lacks discriminative representation ability for clean and occluded face images.
(2) On the basis of the nuclear norm, the additional weighted constraint only yields a marginal gain for occlusion resistance, which indicates that the weighted regularization alone has limited improvement.
(3) When the fusion dictionary strategy is further embedded, the recognition accuracy is substantially improved in all three scenarios. This fully demonstrates that the fusion dictionary effectively compensates for insufficient dictionary completeness under insufficient sampling conditions. Combined with nuclear norm regularization and weighted constraint, it greatly enhances feature representation capability and overall anti-interference performance.

In summary, each designed module plays a complementary and indispensable role. The collaborative combination of nuclear norm, weighted constraint and fusion dictionary jointly guarantees the superior performance of the final proposed algorithm.

## Conclusion

In this work, we propose a novel adaptive robust sparse representation model for face recognition under insufficient sampling and complex interference conditions. The proposed framework integrates three pivotal components: weighted $l_{\text{1}}$ norm constraint, nuclear norm regularization, and fusion dictionary construction. These modules work synergistically to improve feature discriminability, representation stability, and anti-noise robustness. To solve the corresponding optimization problem efficiently, the alternating direction method of multipliers (ADMM) is adopted, and the convergence of the iterative optimization process is theoretically guaranteed. Extensive experiments under clean, noisy and occluded conditions demonstrate that our method outperforms conventional sparse representation and traditional comparison methods.

Furthermore, compared with mainstream deep learning-based schemes, our sparse representation method owns two unique merits. First, it provides high interpretability: sparse coefficients explicitly reflect the contribution of each dictionary atom to feature representation, which is critical for forensic and medical face recognition tasks. Second, it maintains stable performance under limited training samples. Different from deep models that rely on large-scale datasets, the proposed method combines weighted and nuclear norm regularization to alleviate overfitting, and achieves reliable results even with only a small number of training samples per category.

## Supporting information

S1 File Appendix. (DOCX)
