# Supplementary material for: Adaptive robust sparse representation for face recognition based on weighted and fusion dictionary
Source: PLoS One. 2026 Jun 26;21(6):e0351984. doi: 10.1371/journal.pone.0351984 (PMC13309021; doi:10.1371/journal.pone.0351984)
Supplement: S1 File — (DOCX) [file pone.0351984.s001.docx]

**Appendix A**

**Compliance with Dataset Terms of Use**

This research utilizes four public datasets, including the AR Face database, ORL (AT&T) database, Yale Face database, and CMU PIE database. All datasets are used strictly in compliance with their respective terms of use. No personalized identifiable information or private patient data is involved. Therefore, signed consent forms for individual participants are not required. We have included the dataset download link in the manuscript and expressed our gratitude to the data creators in the Acknowledgments section.

**Appendix B**

***Lemma 1:*** Let $X\subset\mathbf{R}^{n}$ be a closed convex set, $F\left(x\right)=H\left(x\right)+G\left(x\right)$, $H\left(x\right):\mathbf{R}^{n}\to\mathbf{R}$ and $G\left(x\right):\mathbf{R}^{n}\to\mathbf{R}$ be convex functions and $G\left(x\right)$ is differentiable. Assume

$x^{*}\text{=}\arg\min_{x} F\left(x\right)$, (a)

then

$x^{*}\in X\text{，}H\left(x\right)-H\left(x^{*}\right)+\left\langle\nabla G\left(x^{*}\right),x-x^{*}\right\rangle\geq0,\text{ }\forall x\in X$ (b)

Proof. If (a) is true, then for any $x\in X$, we have

$\frac{H\left(x_{\alpha}\right)-H\left(x^{*}\right)}{\alpha}+\frac{G\left(x_{\alpha}\right)-G\left(x^{*}\right)}{\alpha}\geq0$, (c)

where

$x_{\alpha}=\left(1-\alpha\right)x^{*}+\alpha x,\text{ }\forall\alpha\in\left(0,1\right]$,

Because $H\left(x\right)$ is convex, it follows that

$H\left(x_{\alpha}\right)\leq\left(1-\alpha\right)H\left(x^{*}\right)+\alpha H\left(x\right)$,

and thus

$H\left(x\right)-H\left(x^{*}\right)\geq\frac{H\left(x_{\alpha}\right)-H\left(x^{*}\right)}{\alpha}\text{, }\forall\alpha\in\left(0,1\right]$,

Substituting the last inequality in the left side of (c), we have

$H\left(x\right)-H\left(x^{*}\right)\text{+}\frac{G\left(x_{\alpha}\right)-G\left(x^{*}\right)}{\alpha}\geq0\text{, }\forall\alpha\in\left(0,1\right]$,

Using $G\left(x_{\alpha}\right)\text{=}G\left(x^{*}+\alpha\left(x-x^{*}\right)\right)$ and letting $\alpha\to0_{\text{+}}$, form the above inequality we get

$H\left(x\right)-H\left(x^{*}\right)+\left\langle\nabla G\left(x^{*}\right),x-x^{*}\right\rangle\geq0\text{, }\forall x\in X$,

Thus (b) follows from (a).

Conversely, since $G\left(x\right)$ is convex, it follows that

$G\left(x_{\alpha}\right)\leq\left(1-\alpha\right)G\left(x^{*}\right)+\alpha G\left(x\right)$,

And it can be rewritten as

$G\left(x_{\alpha}\right)-G\left(x^{*}\right)\leq\alpha\left(G\left(x\right)-G\left(x^{*}\right)\right)$,

Thue, we have

$G\left(x\right)-G\left(x^{*}\right)\geq\frac{G\left(x_{\alpha}\right)-G\left(x^{*}\right)}{\alpha}=\frac{G\left(x^{*}\text{+}\alpha\left(x-x^{*}\right)\right)-G\left(x^{*}\right)}{\alpha}$,

for all $\alpha\in\left(0,1\right]$. Letting $\alpha\to0_{\text{+}}$, we get

$G\left(x\right)-G\left(x^{*}\right)\geq\left\langle\nabla G\left(x^{*}\right),x-x^{*}\right\rangle$,

Substituting it in the left side of (b), we get

$x^{*}\in X\text{，}H\left(x\right)-H\left(x^{*}\right)+G\left(x\right)-G\left(x^{*}\right)\geq0\text{，}\forall x\in X$,

and (a) is true. The proof is complete.

**Appendix C**

The derivation process from Formula (42) and (44) to Formula (45):

$\begin{matrix} \text{-}\left\langle Diag\left(\mathbf{F}^{T}\mathbf{Y}_{2}^{*}\right),\mathbf{x}-\mathbf{x}^{*}\right\rangle-\mu\left\langle Diag\left(\mathbf{F}^{T}\left(\mathbf{J}^{*}-\mathbf{F}Diag\left(\mathbf{x}^{*}\right)\right)\right),\mathbf{x}-\mathbf{x}^{*}\right\rangle\\ \text{+}\left\langle\left(\mathbf{WF}\right)^{T}\mathbf{y}_{1}^{*},\mathbf{x}-\mathbf{x}^{*}\right\rangle\text{+}\mu\left\langle\left(\mathbf{WF}\right)^{T}\left(\hat{\mathbf{e}}^{*}-\mathbf{W}\left(\mathbf{y}-\mathbf{Fx}^{*}\right)\right),\mathbf{x}-\mathbf{x}^{*}\right\rangle\geq0 \end{matrix}$ (42)

$\begin{matrix} \text{-}\left\langle Diag\left(\mathbf{F}^{T}\mathbf{Y}_{2}^{t}\right),\mathbf{x}-\mathbf{x}^{t+1}\right\rangle-\mu\left\langle Diag\left(\mathbf{F}^{T}\left(\mathbf{J}^{t}-\mathbf{F}Diag\left(\mathbf{x}^{t\text{+}1}\right)\right)\right),\mathbf{x}-\mathbf{x}^{t+1}\right\rangle\\ \text{+}\left\langle\left(\mathbf{WF}\right)^{T}\mathbf{y}_{1}^{t},\mathbf{x}-\mathbf{x}^{t+1}\right\rangle\text{+}\mu\left\langle\left(\mathbf{WF}\right)^{T}\left(\hat{\mathbf{e}}^{t}-\mathbf{W}\left(\mathbf{y}-\mathbf{Fx}^{t+1}\right)\right),\mathbf{x}-\mathbf{x}^{t+1}\right\rangle\geq0 \end{matrix}$ (44)

Taking $\mathbf{x}\text{=}\mathbf{x}^{t+1}$ in (42) and $\mathbf{x}\text{=}\mathbf{x}^{*}$ in (44) respectively, we add them together and take the aforementioned relative error into full account ($\mathbf{J}_{e}^{t}\text{=}\mathbf{J}^{t}-\mathbf{J}^{*}$, $\mathbf{x}_{e}^{t}\text{=}\mathbf{x}^{t}-\mathbf{x}^{*}$, $\hat{\mathbf{e}}_{e}^{t}\text{=}\hat{\mathbf{e}}^{t}-\hat{\mathbf{e}}^{*}$, $\mathbf{y}_{1e}^{t}\text{=}\mathbf{y}_{1}^{t}-\mathbf{y}_{1}^{*}$, $\mathbf{Y}_{2e}^{t}\text{=}\mathbf{Y}_{2}^{t}-\mathbf{Y}_{2}^{*}$), then we obtain the following formula (For the detailed derivation process, please refer to the appendix.):

$\begin{matrix} \left\langle\mathbf{Y}_{2e}^{t},\mathbf{F}Diag\left(\mathbf{x}_{e}^{t+1}\right)\right\rangle-\mu\left\langle-\mathbf{J}_{e}^{t}+\mathbf{F}Diag\left(\mathbf{x}_{e}^{t+1}\right),\mathbf{F}Diag\left(\mathbf{x}_{e}^{t+1}\right)\right\rangle\\ -\left\langle\mathbf{y}_{1e}^{t},\mathbf{WFx}_{e}^{t+1}\right\rangle-\mu\left\langle\hat{\mathbf{e}}_{e}^{t}+\mathbf{WFx}_{e}^{t+1},\mathbf{WFx}_{e}^{t+1}\right\rangle\geq0 \end{matrix}$ (45)

**The derivation process is as follow:**

Taking $\mathbf{x}\text{=}\mathbf{x}^{t+1}$ in (42):

$\begin{matrix} \text{-}\left\langle Diag\left(\mathbf{F}^{T}\mathbf{Y}_{2}^{*}\right),\mathbf{x}^{t+1}-\mathbf{x}^{*}\right\rangle-\mu\left\langle Diag\left(\mathbf{F}^{T}\left(\mathbf{J}^{*}-\mathbf{F}Diag\left(\mathbf{x}^{*}\right)\right)\right),\mathbf{x}^{t+1}-\mathbf{x}^{*}\right\rangle\\ \text{+}\left\langle\left(\mathbf{WF}\right)^{T}\mathbf{y}_{1}^{*},\mathbf{x}^{t+1}-\mathbf{x}^{*}\right\rangle\text{+}\mu\left\langle\left(\mathbf{WF}\right)^{T}\left(\hat{\mathbf{e}}^{*}-\mathbf{W}\left(\mathbf{y}-\mathbf{Fx}^{*}\right)\right),\mathbf{x}^{t+1}-\mathbf{x}^{*}\right\rangle\geq0 \end{matrix}$ (a)

Taking $\mathbf{x}\text{=}\mathbf{x}^{*}$ in (44):

$\begin{matrix} \text{-}\left\langle Diag\left(\mathbf{F}^{T}\mathbf{Y}_{2}^{t}\right),\mathbf{x}^{*}-\mathbf{x}^{t+1}\right\rangle-\mu\left\langle Diag\left(\mathbf{F}^{T}\left(\mathbf{J}^{t}-\mathbf{F}Diag\left(\mathbf{x}^{t\text{+}1}\right)\right)\right),\mathbf{x}^{*}-\mathbf{x}^{t+1}\right\rangle\\ \text{+}\left\langle\left(\mathbf{WF}\right)^{T}\mathbf{y}_{1}^{t},\mathbf{x}^{*}-\mathbf{x}^{t+1}\right\rangle\text{+}\mu\left\langle\left(\mathbf{WF}\right)^{T}\left(\hat{\mathbf{e}}^{t}-\mathbf{W}\left(\mathbf{y}-\mathbf{Fx}^{t+1}\right)\right),\mathbf{x}^{*}-\mathbf{x}^{t+1}\right\rangle\geq0 \end{matrix}$ (b)

The first group:

$\begin{matrix} \text{-}\left\langle Diag\left(\mathbf{F}^{T}\mathbf{Y}_{2}^{*}\right),\mathbf{x}^{t+1}-\mathbf{x}^{*}\right\rangle\text{-}\left\langle Diag\left(\mathbf{F}^{T}\mathbf{Y}_{2}^{t}\right),\mathbf{x}^{*}-\mathbf{x}^{t+1}\right\rangle\\ =\left\langle Diag\left(\mathbf{F}^{T}\left(\mathbf{Y}_{2}^{t}-\mathbf{Y}_{2}^{*}\right)\right),\mathbf{x}^{t+1}-\mathbf{x}^{*}\right\rangle\\ =\left\langle Diag\left(\mathbf{F}^{T}\left(\mathbf{Y}_{2e}^{t}\right)\right),\mathbf{x}_{e}^{t+1}\right\rangle\\ =\left\langle\mathbf{F}^{T}\mathbf{Y}_{2e}^{t},Diag\left(\mathbf{x}_{e}^{t+1}\right)\right\rangle\\ =\left\langle\mathbf{Y}_{2e}^{t},\mathbf{F}Diag\left(\mathbf{x}_{e}^{t+1}\right)\right\rangle\end{matrix}$ (c)

The second group:

$\begin{matrix} -\mu\left\langle Diag\left(\mathbf{F}^{T}\left(\mathbf{J}^{*}-\mathbf{F}Diag\left(\mathbf{x}^{*}\right)\right)\right),\mathbf{x}^{t+1}-\mathbf{x}^{*}\right\rangle-\mu\left\langle Diag\left(\mathbf{F}^{T}\left(\mathbf{J}^{t}-\mathbf{F}Diag\left(\mathbf{x}^{t\text{+}1}\right)\right)\right),\mathbf{x}^{*}-\mathbf{x}^{t+1}\right\rangle\\ =-\mu\left\langle Diag\left(\mathbf{F}^{T}\left(\mathbf{J}^{*}-\mathbf{J}^{t}-\mathbf{F}Diag\left(\mathbf{x}^{*}\right)+\mathbf{F}Diag\left(\mathbf{x}^{t\text{+}1}\right)\right)\right),\mathbf{x}^{t+1}-\mathbf{x}^{*}\right\rangle\\ =-\mu\left\langle Diag\left(\mathbf{F}^{T}\left(-\mathbf{J}_{e}^{t}+\mathbf{F}Diag\left(\mathbf{x}^{t+1}-\mathbf{x}^{*}\right)\right)\right),\mathbf{x}^{t+1}-\mathbf{x}^{*}\right\rangle\\ =-\mu\left\langle Diag\left(\mathbf{F}^{T}\left(-\mathbf{J}_{e}^{t}+\mathbf{F}Diag\left(\mathbf{x}_{e}^{t\text{+}1}\right)\right)\right),\mathbf{x}_{e}^{t\text{+}1}\right\rangle\\ =-\mu\left\langle\mathbf{F}^{T}\left(-\mathbf{J}_{e}^{t}+\mathbf{F}Diag\left(\mathbf{x}_{e}^{t\text{+}1}\right)\right),Diag\left(\mathbf{x}_{e}^{t\text{+}1}\right)\right\rangle\\ =-\mu\left\langle-\mathbf{J}_{e}^{t}+\mathbf{F}Diag\left(\mathbf{x}_{e}^{t\text{+}1}\right),\mathbf{F}Diag\left(\mathbf{x}_{e}^{t\text{+}1}\right)\right\rangle\end{matrix}$ (d)

The third group:

$\begin{matrix} \left\langle\left(\mathbf{WF}\right)^{T}\mathbf{y}_{1}^{*},\mathbf{x}^{t+1}-\mathbf{x}^{*}\right\rangle\text{+}\left\langle\left(\mathbf{WF}\right)^{T}\mathbf{y}_{1}^{t},\mathbf{x}^{*}-\mathbf{x}^{t+1}\right\rangle\\ =\left\langle\left(\mathbf{WF}\right)^{T}\left(\mathbf{y}_{1}^{*}-\mathbf{y}_{1}^{t}\right),\mathbf{x}^{t+1}-\mathbf{x}^{*}\right\rangle\\ =-\left\langle\left(\mathbf{WF}\right)^{T}\left(\mathbf{y}_{1e}^{t}\right),\mathbf{x}_{e}^{t+1}\right\rangle\\ =-\left\langle\mathbf{y}_{1e}^{t},\mathbf{WFx}_{e}^{t+1}\right\rangle\end{matrix}$ (e)

The fourth group:

$\begin{matrix} \mu\left\langle\left(\mathbf{WF}\right)^{T}\left(\hat{\mathbf{e}}^{*}-\mathbf{W}\left(\mathbf{y}-\mathbf{Fx}^{*}\right)\right),\mathbf{x}^{t+1}-\mathbf{x}^{*}\right\rangle\text{+}\mu\left\langle\left(\mathbf{WF}\right)^{T}\left(\hat{\mathbf{e}}^{t}-\mathbf{W}\left(\mathbf{y}-\mathbf{Fx}^{t+1}\right)\right),\mathbf{x}^{*}-\mathbf{x}^{t+1}\right\rangle\\ =\mu\left\langle\left(\mathbf{WF}\right)^{T}\left(\hat{\mathbf{e}}^{*}-\mathbf{W}\left(\mathbf{y}-\mathbf{Fx}^{*}\right)-\hat{\mathbf{e}}^{t}+\mathbf{W}\left(\mathbf{y}-\mathbf{Fx}^{t+1}\right)\right),\mathbf{x}^{t+1}-\mathbf{x}^{*}\right\rangle\\ =\mu\left\langle\left(\mathbf{WF}\right)^{T}\left(\hat{\mathbf{e}}^{*}-\hat{\mathbf{e}}^{t}+\mathbf{WFx}^{*}-\mathbf{WFx}^{t+1}\right),\mathbf{x}^{t+1}-\mathbf{x}^{*}\right\rangle\\ =-\mu\left\langle\left(\mathbf{WF}\right)^{T}\left(\hat{\mathbf{e}}_{e}^{t}+\mathbf{WFx}_{e}^{t+1}\right),\mathbf{x}_{e}^{t+1}\right\rangle\\ =-\mu\left\langle\left(\hat{\mathbf{e}}_{e}^{t}+\mathbf{WFx}_{e}^{t+1}\right),\mathbf{WFx}_{e}^{t+1}\right\rangle\end{matrix}$ (f)

Summarized result is

$\begin{matrix} \left\langle\mathbf{Y}_{2e}^{t},\mathbf{F}Diag\left(\mathbf{x}_{e}^{t+1}\right)\right\rangle-\mu\left\langle-\mathbf{J}_{e}^{t}+\mathbf{F}Diag\left(\mathbf{x}_{e}^{t+1}\right),\mathbf{F}Diag\left(\mathbf{x}_{e}^{t+1}\right)\right\rangle\\ -\left\langle\mathbf{y}_{1e}^{t},\mathbf{WFx}_{e}^{t+1}\right\rangle-\mu\left\langle\hat{\mathbf{e}}_{e}^{t}+\mathbf{WFx}_{e}^{t+1},\mathbf{WFx}_{e}^{t+1}\right\rangle\geq0 \end{matrix}$ (45)

**Appendix D**

The intermediate explanation from the individual monotonicity inequalities (equations (45) – (47)) to the overall inequality (equation 48)

$\begin{matrix} \left\langle\mathbf{Y}_{2e}^{t}\text{，}\mathbf{F}Diag\left(\mathbf{x}_{e}^{t+1}\right)\right\rangle-\mu\left\langle-\mathbf{J}_{e}^{t}+\mathbf{F}Diag\left(\mathbf{x}_{e}^{t+1}\right),\mathbf{F}Diag\left(\mathbf{x}_{e}^{t+1}\right)\right\rangle\\ -\left\langle\mathbf{y}_{1e}^{t},\mathbf{WFx}_{e}^{t+1}\right\rangle-\mu\left\langle\hat{\mathbf{e}}_{e}^{t}+\mathbf{WFx}_{e}^{t+1},\mathbf{WFx}_{e}^{t+1}\right\rangle\geq0 \end{matrix}$ (45)

Similarly, we have

$\left\langle\mathbf{Y}_{2e}^{t},-\mathbf{J}_{e}^{t\text{+}1}\right\rangle\text{+}\mu\left\langle\mathbf{F}Diag\left(\mathbf{x}_{e}^{t+1}\right)-\mathbf{J}_{e}^{t\text{+}1},\mathbf{J}_{e}^{t\text{+}1}\right\rangle\geq0$, (46) $\left\langle\mathbf{y}_{1e}^{t},-\hat{\mathbf{e}}_{e}^{t\text{+}1}\right\rangle-\mu\left\langle\hat{\mathbf{e}}_{e}^{t\text{+}1}+\mathbf{WFx}_{e}^{t+1},\hat{\mathbf{e}}_{e}^{t\text{+}1}\right\rangle\geq0$, (47)

Summing (45)-(47) together follows that

$\begin{matrix} -\left\langle\mathbf{y}_{1e}^{t}\text{，}\hat{\mathbf{e}}_{e}^{t\text{+}1}+\mathbf{WFx}_{e}^{t+1}\right\rangle-\left\langle\mathbf{Y}_{2e}^{t},\mathbf{J}_{e}^{t\text{+}1}-\mathbf{F}Diag\left(\mathbf{x}_{e}^{t+1}\right)\right\rangle\\ \geq\mu\left\|\mathbf{WFx}_{e}^{t+1}+\hat{\mathbf{e}}_{e}^{t\text{+}1}\right\|_{2}^{2}+\mu\left\langle\mathbf{WFx}_{e}^{t+1},\hat{\mathbf{e}}_{e}^{t}-\hat{\mathbf{e}}_{e}^{t\text{+}1}\right\rangle\\ +\mu\left\|\mathbf{F}Diag\left(\mathbf{x}_{e}^{t+1}\right)-\mathbf{J}_{e}^{t+1}\right\|_{F}^{2}+\mu\left\langle\mathbf{F}Diag\left(\mathbf{x}_{e}^{t+1}\right),\mathbf{J}_{e}^{t\text{+}1}-\mathbf{J}_{e}^{t}\right\rangle\end{matrix}$ (48)

**The derivation process is as follow:**

Summing the (45) – (47) three formulas

$\begin{matrix} \left\langle\mathbf{Y}_{2e}^{t},\mathbf{F}Diag\left(\mathbf{x}_{e}^{t+1}\right)\right\rangle+\left\langle\mathbf{Y}_{2e}^{t},-\mathbf{J}_{e}^{t\text{+}1}\right\rangle-\left\langle\mathbf{y}_{1e}^{t},\mathbf{WFx}_{e}^{t+1}\right\rangle+\left\langle\mathbf{y}_{1e}^{t},-\hat{\mathbf{e}}_{e}^{t\text{+}1}\right\rangle\\ -\mu\left[\left\langle-\mathbf{J}_{e}^{t}+\mathbf{F}Diag\left(\mathbf{x}_{e}^{t+1}\right),\mathbf{F}Diag\left(\mathbf{x}_{e}^{t+1}\right)\right\rangle+\mu\left\langle\hat{\mathbf{e}}_{e}^{t}+\mathbf{WFx}_{e}^{t+1},\mathbf{WFx}_{e}^{t+1}\right\rangle\right] \\ -\mu\left[-\left\langle\mathbf{F}Diag\left(\mathbf{x}_{e}^{t+1}\right)-\mathbf{J}_{e}^{t\text{+}1},\mathbf{J}_{e}^{t\text{+}1}\right\rangle+\mu\left\langle\hat{\mathbf{e}}_{e}^{t\text{+}1}+\mathbf{WFx}_{e}^{t+1},\hat{\mathbf{e}}_{e}^{t\text{+}1}\right\rangle\right]\geq0 \end{matrix}$ (a)

Combine the terms without μ while reorganizing the terms containing μ

$\begin{matrix} -\left\langle\mathbf{y}_{1e}^{t},\mathbf{WFx}_{e}^{t+1}+\hat{\mathbf{e}}_{e}^{t\text{+}1}\right\rangle-\left\langle\mathbf{Y}_{2e}^{t},\mathbf{J}_{e}^{t\text{+}1}-\mathbf{F}Diag\left(\mathbf{x}_{e}^{t+1}\right)\right\rangle\\ -\mu\left[\left\langle-\mathbf{J}_{e}^{t},\mathbf{F}Diag\left(\mathbf{x}_{e}^{t+1}\right)\right\rangle+\left\langle\mathbf{F}Diag\left(\mathbf{x}_{e}^{t+1}\right),\mathbf{F}Diag\left(\mathbf{x}_{e}^{t+1}\right)\right\rangle+\left\langle\hat{\mathbf{e}}_{e}^{t},\mathbf{WFx}_{e}^{t+1}\right\rangle+\left\langle\mathbf{WFx}_{e}^{t+1},\mathbf{WFx}_{e}^{t+1}\right\rangle\right] \\ -\mu\left[-\left\langle\mathbf{F}Diag\left(\mathbf{x}_{e}^{t+1}\right),\mathbf{J}_{e}^{t\text{+}1}\right\rangle+\left\langle\mathbf{J}_{e}^{t\text{+}1},\mathbf{J}_{e}^{t\text{+}1}\right\rangle+\left\langle\hat{\mathbf{e}}_{e}^{t\text{+}1},\hat{\mathbf{e}}_{e}^{t\text{+}1}\right\rangle+\left\langle\mathbf{WFx}_{e}^{t+1},\hat{\mathbf{e}}_{e}^{t\text{+}1}\right\rangle\right]\geq0 \end{matrix}$ (b)

$\begin{matrix} -\left\langle\mathbf{y}_{1e}^{t},\mathbf{WFx}_{e}^{t+1}+\hat{\mathbf{e}}_{e}^{t\text{+}1}\right\rangle-\left\langle\mathbf{Y}_{2e}^{t},\mathbf{J}_{e}^{t\text{+}1}-\mathbf{F}Diag\left(\mathbf{x}_{e}^{t+1}\right)\right\rangle\\ -\mu\left[\left\langle-\mathbf{J}_{e}^{t},\mathbf{F}Diag\left(\mathbf{x}_{e}^{t+1}\right)\right\rangle+\left\|\mathbf{F}Diag\left(\mathbf{x}_{e}^{t+1}\right)\right\|_{2}^{2}+\left\langle\hat{\mathbf{e}}_{e}^{t},\mathbf{WFx}_{e}^{t+1}\right\rangle+\left\|\mathbf{WFx}_{e}^{t+1}\right\|_{2}^{2}\right] \\ -\mu\left[-\left\langle\mathbf{F}Diag\left(\mathbf{x}_{e}^{t+1}\right),\mathbf{J}_{e}^{t\text{+}1}\right\rangle+\left\|\mathbf{J}_{e}^{t\text{+}1}\right\|_{2}^{2}+\left\|\hat{\mathbf{e}}_{e}^{t\text{+}1}\right\|_{2}^{2}+\left\langle\mathbf{WFx}_{e}^{t+1},\hat{\mathbf{e}}_{e}^{t\text{+}1}\right\rangle\right]\geq0 \end{matrix}$ (c)

$\begin{matrix} -\left\langle\mathbf{y}_{1e}^{t},\mathbf{WFx}_{e}^{t+1}+\hat{\mathbf{e}}_{e}^{t\text{+}1}\right\rangle-\left\langle\mathbf{Y}_{2e}^{t},\mathbf{J}_{e}^{t\text{+}1}-\mathbf{F}Diag\left(\mathbf{x}_{e}^{t+1}\right)\right\rangle\\ -\mu\left[\left\|\mathbf{WFx}_{e}^{t+1}\right\|_{2}^{2}+\left\|\hat{\mathbf{e}}_{e}^{t\text{+}1}\right\|_{2}^{2}+\left\langle\hat{\mathbf{e}}_{e}^{t},\mathbf{WFx}_{e}^{t+1}\right\rangle+\left\langle\mathbf{WFx}_{e}^{t+1},\hat{\mathbf{e}}_{e}^{t\text{+}1}\right\rangle+\right] \\ -\mu\left[\left\|\mathbf{F}Diag\left(\mathbf{x}_{e}^{t+1}\right)\right\|_{2}^{2}+\left\|\mathbf{J}_{e}^{t\text{+}1}\right\|_{2}^{2}+\left\langle-\mathbf{J}_{e}^{t},\mathbf{F}Diag\left(\mathbf{x}_{e}^{t+1}\right)\right\rangle-\left\langle\mathbf{F}Diag\left(\mathbf{x}_{e}^{t+1}\right),\mathbf{J}_{e}^{t\text{+}1}\right\rangle\right]\geq0 \end{matrix}$ (d)

$\begin{matrix} -\left\langle\mathbf{y}_{1e}^{t},\mathbf{WFx}_{e}^{t+1}+\hat{\mathbf{e}}_{e}^{t\text{+}1}\right\rangle-\left\langle\mathbf{Y}_{2e}^{t},\mathbf{J}_{e}^{t\text{+}1}-\mathbf{F}Diag\left(\mathbf{x}_{e}^{t+1}\right)\right\rangle\\ -\mu\left[\left\|\mathbf{WFx}_{e}^{t+1}+\hat{\mathbf{e}}_{e}^{t\text{+}1}\right\|_{2}^{2}+\left\langle\hat{\mathbf{e}}_{e}^{t},\mathbf{WFx}_{e}^{t+1}\right\rangle-\left\langle\mathbf{WFx}_{e}^{t+1},\hat{\mathbf{e}}_{e}^{t\text{+}1}\right\rangle+\right] \\ -\mu\left[\left\|\mathbf{F}Diag\left(\mathbf{x}_{e}^{t+1}\right)-\mathbf{J}_{e}^{t\text{+}1}\right\|_{2}^{2}-\left\langle\mathbf{J}_{e}^{t},\mathbf{F}Diag\left(\mathbf{x}_{e}^{t+1}\right)\right\rangle+\left\langle\mathbf{F}Diag\left(\mathbf{x}_{e}^{t+1}\right),\mathbf{J}_{e}^{t\text{+}1}\right\rangle\right]\geq0 \end{matrix}$ (e)

$\begin{matrix} -\left\langle\mathbf{y}_{1e}^{t},\mathbf{WFx}_{e}^{t+1}+\hat{\mathbf{e}}_{e}^{t\text{+}1}\right\rangle-\left\langle\mathbf{Y}_{2e}^{t},\mathbf{J}_{e}^{t\text{+}1}-\mathbf{F}Diag\left(\mathbf{x}_{e}^{t+1}\right)\right\rangle\\ -\mu\left[\left\|\mathbf{WFx}_{e}^{t+1}+\hat{\mathbf{e}}_{e}^{t\text{+}1}\right\|_{2}^{2}+\left\langle\hat{\mathbf{e}}_{e}^{t}-\hat{\mathbf{e}}_{e}^{t\text{+}1},\mathbf{WFx}_{e}^{t+1}\right\rangle+\right] \\ -\mu\left[\left\|\mathbf{F}Diag\left(\mathbf{x}_{e}^{t+1}\right)-\mathbf{J}_{e}^{t\text{+}1}\right\|_{2}^{2}+\left\langle\mathbf{F}Diag\left(\mathbf{x}_{e}^{t+1}\right),\mathbf{J}_{e}^{t\text{+}1}-\mathbf{J}_{e}^{t}\right\rangle\right]\geq0 \end{matrix}$ (f)

So, the equation (48) can be obtained as follow,

$\begin{matrix} -\left\langle\mathbf{y}_{1e}^{t},\mathbf{WFx}_{e}^{t+1}+\hat{\mathbf{e}}_{e}^{t\text{+}1}\right\rangle-\left\langle\mathbf{Y}_{2e}^{t},\mathbf{J}_{e}^{t\text{+}1}-\mathbf{F}Diag\left(\mathbf{x}_{e}^{t+1}\right)\right\rangle\\ \geq\mu\left\|\mathbf{WFx}_{e}^{t+1}+\hat{\mathbf{e}}_{e}^{t\text{+}1}\right\|_{2}^{2}+\mu\left\langle\hat{\mathbf{e}}_{e}^{t}-\hat{\mathbf{e}}_{e}^{t\text{+}1},\mathbf{WFx}_{e}^{t+1}\right\rangle\\ +\mu\left\|\mathbf{F}Diag\left(\mathbf{x}_{e}^{t+1}\right)-\mathbf{J}_{e}^{t\text{+}1}\right\|_{2}^{2}+\mu\left\langle\mathbf{F}Diag\left(\mathbf{x}_{e}^{t+1}\right),\mathbf{J}_{e}^{t\text{+}1}-\mathbf{J}_{e}^{t}\right\rangle\end{matrix}$ (48)

**Appendix E**

The derivation process from Formula (51) and (52) to Formula (53):

$\lambda\left(\left\|\mathbf{J}\right\|_{*}-\left\|\mathbf{J}^{t\text{+1}}\right\|_{*}\right)+\left\langle\mathbf{Y}_{2}^{t},\mathbf{J}-\mathbf{J}^{t+1}\right\rangle+\mu\left\langle\mathbf{J}^{t+1}-\mathbf{F}Diag\left(\mathbf{x}^{t+1}\right),\mathbf{J}-\mathbf{J}^{t+1}\right\rangle\geq0$ (51)

$\lambda\left(\left\|\mathbf{J}\right\|_{*}-\left\|\mathbf{J}^{t}\right\|_{*}\right)+\left\langle\mathbf{Y}_{2}^{t\text{-}1},\mathbf{J}-\mathbf{J}^{t}\right\rangle+\mu\left\langle\mathbf{J}^{t}-\mathbf{F}Diag\left(\mathbf{x}^{t}\right),\mathbf{J}-\mathbf{J}^{t}\right\rangle\geq0$ (52)

By taking $\mathbf{J}\text{=}\mathbf{J}^{t}$ in (51) and $\mathbf{J}\text{=}\mathbf{J}^{t+1}$ in (52) respectively, and we add them together and take the aforementioned relative error into full account ($\mathbf{J}_{e}^{t}\text{=}\mathbf{J}^{t}-\mathbf{J}^{*}$, $\mathbf{x}_{e}^{t}\text{=}\mathbf{x}^{t}-\mathbf{x}^{*}$, $\hat{\mathbf{e}}_{e}^{t}\text{=}\hat{\mathbf{e}}^{t}-\hat{\mathbf{e}}^{*}$, $\mathbf{y}_{1e}^{t}\text{=}\mathbf{y}_{1}^{t}-\mathbf{y}_{1}^{*}$, $\mathbf{Y}_{2e}^{t}\text{=}\mathbf{Y}_{2}^{t}-\mathbf{Y}_{2}^{*}$), then we obtain the following formula (For the detailed derivation process, please refer to the appendix.):

$\left\langle\mathbf{Y}_{2e}^{t-1}\text{-}\mathbf{Y}_{2e}^{t},\mathbf{J}_{e}^{t+1}-\mathbf{J}_{e}^{t}\right\rangle-\mu\left\|\mathbf{J}_{e}^{t+1}-\mathbf{J}_{e}^{t}\right\|_{F}^{2}-\mu\left\langle\mathbf{F}Diag\left(\mathbf{x}_{e}^{t}-\mathbf{x}_{e}^{t+1}\right),\mathbf{J}_{e}^{t+1}-\mathbf{J}_{e}^{t}\right\rangle\geq0$ (53)

**The derivation process is as follow:**

Taking $\mathbf{J}\text{=}\mathbf{J}^{t}$ in (51):

$\lambda\left(\left\|\mathbf{J}^{t}\right\|_{*}-\left\|\mathbf{J}^{t\text{+1}}\right\|_{*}\right)+\left\langle\mathbf{Y}_{2}^{t},\mathbf{J}^{t}-\mathbf{J}^{t+1}\right\rangle+\mu\left\langle\mathbf{J}^{t+1}-\mathbf{F}Diag\left(\mathbf{x}^{t+1}\right),\mathbf{J}^{t}-\mathbf{J}^{t+1}\right\rangle\geq0$ (a)

Taking $\mathbf{J}\text{=}\mathbf{J}^{t+1}$ in (52):

$\lambda\left(\left\|\mathbf{J}^{t+1}\right\|_{*}-\left\|\mathbf{J}^{t}\right\|_{*}\right)+\left\langle\mathbf{Y}_{2}^{t\text{-}1},\mathbf{J}^{t+1}-\mathbf{J}^{t}\right\rangle+\mu\left\langle\mathbf{J}^{t}-\mathbf{F}Diag\left(\mathbf{x}^{t}\right),\mathbf{J}^{t+1}-\mathbf{J}^{t}\right\rangle\geq0$ (b)

The first group:

$\lambda\left(\left\|\mathbf{J}^{t}\right\|_{*}-\left\|\mathbf{J}^{t\text{+1}}\right\|_{*}\right)+\lambda\left(\left\|\mathbf{J}^{t+1}\right\|_{*}-\left\|\mathbf{J}^{t}\right\|_{*}\right)=0$ (c)

The second group:

$\begin{matrix} \left\langle\mathbf{Y}_{2}^{t},\mathbf{J}^{t}-\mathbf{J}^{t+1}\right\rangle+\left\langle\mathbf{Y}_{2}^{t\text{-}1},\mathbf{J}^{t+1}-\mathbf{J}^{t}\right\rangle\\ =-\left\langle\mathbf{Y}_{2}^{t},\mathbf{J}^{t+1}-\mathbf{J}^{t}\right\rangle+\left\langle\mathbf{Y}_{2}^{t\text{-}1},\mathbf{J}^{t+1}-\mathbf{J}^{t}\right\rangle\\ =\left\langle\mathbf{Y}_{2}^{t\text{-}1}-\mathbf{Y}_{2}^{t},\mathbf{J}^{t+1}-\mathbf{J}^{t}\right\rangle\\ =\left\langle\mathbf{Y}_{2}^{t\text{-}1}-\mathbf{Y}_{2}^{*}-\mathbf{Y}_{2}^{t}+\mathbf{Y}_{2}^{*},\mathbf{J}^{t+1}-\mathbf{J}^{*}-\mathbf{J}^{t}+\mathbf{J}^{*}\right\rangle\\ =\left\langle\mathbf{Y}_{2e}^{t\text{-}1}-\mathbf{Y}_{2e}^{t},\mathbf{J}_{e}^{t+1}-\mathbf{J}_{e}^{t}\right\rangle\end{matrix}$ (d)

The third group:

$\begin{matrix} \mu\left\langle\mathbf{J}^{t+1}-\mathbf{F}Diag\left(\mathbf{x}^{t+1}\right),\mathbf{J}^{t}-\mathbf{J}^{t+1}\right\rangle+\mu\left\langle\mathbf{J}^{t}-\mathbf{F}Diag\left(\mathbf{x}^{t}\right),\mathbf{J}^{t+1}-\mathbf{J}^{t}\right\rangle\\ =\mu\left\langle\mathbf{J}^{t+1},\mathbf{J}^{t}-\mathbf{J}^{t+1}\right\rangle+\mu\left\langle\mathbf{J}^{t},\mathbf{J}^{t+1}-\mathbf{J}^{t}\right\rangle-\mu\left\langle\mathbf{F}Diag\left(\mathbf{x}^{t+1}\right),\mathbf{J}^{t}-\mathbf{J}^{t+1}\right\rangle-\mu\left\langle\mathbf{F}Diag\left(\mathbf{x}^{t}\right),\mathbf{J}^{t+1}-\mathbf{J}^{t}\right\rangle\\ =\mu\left\langle\mathbf{J}^{t}-\mathbf{J}^{t+1},\mathbf{J}^{t+1}-\mathbf{J}^{t}\right\rangle-\mu\left\langle\mathbf{F}Diag\left(\mathbf{x}^{t}-\mathbf{x}^{t+1}\right),\mathbf{J}^{t+1}-\mathbf{J}^{t}\right\rangle\\ =-\mu\left\|\mathbf{J}^{t+1}-\mathbf{J}^{t}\right\|_{F}^{2}-\mu\left\langle\mathbf{F}Diag\left(\mathbf{x}^{t}-\mathbf{x}^{t+1}\right),\mathbf{J}^{t+1}-\mathbf{J}^{t}\right\rangle\\ =-\mu\left\|\mathbf{J}^{t+1}-\mathbf{J}^{*}-\mathbf{J}^{t}+\mathbf{J}^{*}\right\|_{F}^{2}-\mu\left\langle\mathbf{F}Diag\left(\mathbf{x}^{t}-\mathbf{x}^{*}-\mathbf{x}^{t+1}+\mathbf{x}^{*}\right),\mathbf{J}^{t+1}-\mathbf{J}^{*}-\mathbf{J}^{t}+\mathbf{J}^{*}\right\rangle\\ =-\mu\left\|\mathbf{J}_{e}^{t+1}-\mathbf{J}_{e}^{t}\right\|_{F}^{2}-\mu\left\langle\mathbf{F}Diag\left(\mathbf{x}_{e}^{t}-\mathbf{x}_{e}^{t+1}\right),\mathbf{J}_{e}^{t+1}-\mathbf{J}_{e}^{t}\right\rangle\end{matrix}$ (e)

Summarized result is

$\left\langle\mathbf{Y}_{2e}^{t-1}\text{-}\mathbf{Y}_{2e}^{t},\mathbf{J}_{e}^{t+1}-\mathbf{J}_{e}^{t}\right\rangle-\mu\left\|\mathbf{J}_{e}^{t+1}-\mathbf{J}_{e}^{t}\right\|_{F}^{2}-\mu\left\langle\mathbf{F}Diag\left(\mathbf{x}_{e}^{t}-\mathbf{x}_{e}^{t+1}\right),\mathbf{J}_{e}^{t+1}-\mathbf{J}_{e}^{t}\right\rangle\geq0$ (53)
